# Digoxin Alleviates Osteoarthritis by Inhibiting Macrophage Extracellular Trap Formation via Disruption of the LSP1‐SOD1 Interaction

**DOI:** 10.1002/advs.78097

**Published:** 2026-09-30

**Authors:** Yankai Pan, Xinzhe Feng, Chuanju Liu, Jianfei Tong, Zhi Li, Chenxu Liu, Yutong Dong, Chen Meng, Yihong Xu, Weidong Xu

**Affiliations:** ^1^ Department of Joint Bone Disease Surgery Changhai Hospital Naval Medical University Shanghai China; ^2^ Department of Orthopaedics and Rehabilitation Yale University School of Medicine New Haven Connecticut USA

**Keywords:** digoxin, LSP1, macrophage extracellular traps, osteoarthritis, SOD1

## Abstract

Osteoarthritis (OA) is a chronic degenerative joint disease and a leading cause of disability worldwide, yet no disease‐modifying therapies are currently available. The role of synovial macrophages in OA pathogenesis remains incompletely understood, and the molecular mechanisms by which they drive cartilage destruction have not been fully elucidated. By integrating human OA microarrays, synovial single‐cell RNA‐seq, and multi‐level validation, we identified lymphocyte‐specific protein 1 (LSP1) as a macrophage‐specific hub gene upregulated in OA. Mechanistically, LSP1 directly binds to and inhibits superoxide dismutase 1 (SOD1), inducing a redox imbalance that concurrently activates the NADPH oxidase (NOX) and mitochondrial reactive oxygen species (mtROS) pathways. This cascade drives the release of macrophage extracellular traps (METs), which directly damage chondrocytes and accelerate OA progression. Virtual screening of the DrugBank database identified the cardiac glycoside digoxin as a compound that competitively binds LSP1, restores SOD1 activity, and suppresses the NOX/mtROS‐METs axis. This study defines a novel LSP1‐SOD1‐METs pathogenic axis in OA and nominates digoxin as a repurposable therapeutic candidate. Unlike its previously reported cartilage‐targeting mechanism, digoxin alleviates OA by suppressing synovial inflammation via LSP1 targeting, highlighting its dual‐target potential for concurrent modulation of synovial and cartilage pathology.

## Graphical abstract

Synovial macrophages in osteoarthritis upregulate LSP1, which directly binds and inhibits SOD1, triggering a redox imbalance that activates both NOX and mitochondrial ROS pathways. This cascade drives the release of macrophage extracellular traps (METs), causing chondrocyte damage and disease progression. The cardiac glycoside digoxin competitively disrupts the LSP1–SOD1 interaction, restores redox homeostasis, and suppresses METs formation, highlighting a repurposable therapeutic strategy targeting synovial inflammation in osteoarthritis.

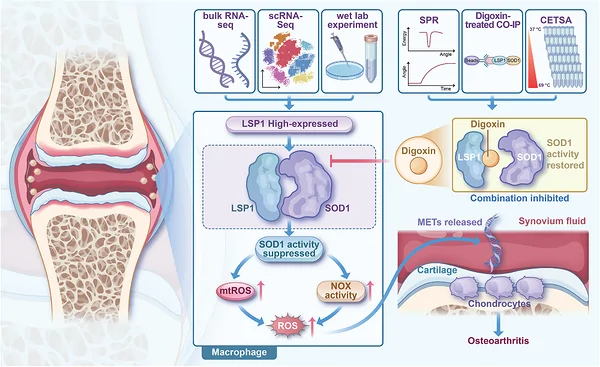

## 1 Introduction

Osteoarthritis (OA), a chronic degenerative joint disease and a leading cause of joint pain and functional impairment, is increasing in prevalence with the aging of the global population and imposing a substantial global disease burden [1, 2]. Currently, no clinical therapies can fully reverse cartilage damage. Therefore, elucidating the pathological mechanisms of OA and identifying potential therapeutic targets are crucial for improving patient outcomes. Traditionally, OA has been viewed as a primarily mechanical disorder driven by cartilage wear or injury [3]. However, recent research has increasingly highlighted the pivotal role of inflammation and immune system alterations in OA pathogenesis [4, 5]. Growing evidence indicates that both systemic and localized immune‐inflammatory responses, particularly chronic low‐grade synovitis, serve as central drivers of joint degeneration, pain, and functional impairment [6, 7].

Macrophages represent the predominant leukocyte population in the synovial joint fluid of OA patients, constituting approximately 40% of the total immune cells in the OA synovial membrane [8]. While essential for homeostasis under physiological conditions [9], their aberrant activation in OA exacerbates joint damage [10]. However, the precise molecular mechanisms by which synovial macrophages drive cartilage destruction in OA remain incompletely understood.

Emerging evidence has implicated extracellular traps in the pathogenesis of various inflammatory diseases. Neutrophil extracellular traps (NETs)—web‐like structures composed of DNA, histones, and granule proteins—are released by neutrophils and contribute to tissue damage in conditions including rheumatoid arthritis, atherosclerosis, and cancer [11, 12, 13]. The formation of NETs is critically dependent on the activation of NADPH oxidase (NOX) and the generation of mitochondrial reactive oxygen species (mtROS) [14]. More recently, macrophages have been shown to release morphologically analogous structures termed macrophage extracellular traps (METs), which are implicated in the pathology of atherosclerosis, acute kidney injury, and certain malignancies [15, 16, 17, 18, 19, 20, 21]. Recent studies have begun to elucidate two major pathogenic functions of METs: their involvement in sterile inflammation [22, 23, 24] and their contribution to tissue remodeling and fibrosis [25, 26]. Given that OA is characterized by both chronic low‐grade synovitis—a prototypical sterile inflammatory condition—and progressive cartilage degradation, we hypothesized that METs may likewise participate in OA pathogenesis. However, whether METs accumulate in OA joints and directly contribute to chondrocyte damage has remained unexplored.

Oxidative stress and reactive oxygen species (ROS) play a critical role in OA pathogenesis. Excessive ROS production, primarily derived from NOX enzymes and mitochondrial dysfunction, has been documented in OA synovium and cartilage, where it promotes chondrocyte senescence, apoptosis, and matrix degradation [27, 28, 29]. Notably, the NOX/mtROS axis is not only essential for NETs formation but also contributes to the inflammatory synovial microenvironment in OA [30]. Given the structural and functional parallels between NETs and METs, we reasoned that a similar NOX/mtROS‐dependent mechanism might underpin METs formation in OA macrophages. However, the upstream regulators linking oxidative stress to METs release in the OA joint remain largely unexplored.

Identifying the upstream regulators of this pathogenic cascade in OA macrophages, however, poses a fundamental challenge. The synovial microenvironment is a complex ecosystem in which pathogenic drivers are embedded within interconnected molecular networks, rather than acting in isolation. Within such networks, certain “hub genes” occupy central positions, integrating diverse inflammatory signals and coordinating multiple downstream pathways through a single node. Their dysregulation in a disease‐relevant cell type can amplify pathological signals across multiple axes, whereas their therapeutic targeting offers the potential to modulate several pathogenic pathways with a single intervention. Identifying macrophage‐enriched hub genes that are functionally positioned within inflammatory and matrix‐remodeling networks therefore represents a powerful strategy for uncovering novel mechanistic drivers with high translational relevance—one that transcends the limitations of conventional candidate‐gene selection. We therefore aimed to systematically nominate such genes in OA through an unbiased, integrative transcriptomic approach.

Current pharmacological management of OA is primarily limited to symptom control through intra‐articular injections (e.g., hyaluronic acid, corticosteroids) and systemic analgesics (e.g., NSAIDs). These interventions fail to modify disease progression, and their long‐term efficacy and safety remain contentious [31, 32]. Consequently, there is an urgent need to identify therapeutic strategies that can slow or halt OA progression. In this context, the “drug repurposing” strategy—applying FDA‐approved drugs to new indications—is highly attractive as it can drastically reduce the time and cost of drug development. The translational appeal of repurposing further lies in the established knowledge accompanying approved drugs—their pharmacokinetic profiles, safety records, and manufacturing infrastructure are already in place, substantially lowering the barriers to clinical application. Moreover, recent advances in computational screening have enabled the systematic identification of repurposing candidates by leveraging mechanistic insights from multi‐omics data, allowing researchers to match existing drugs to newly discovered pathogenic targets with greater precision than traditional empirical screening.

In this study, we aimed to identify key macrophage‐derived drivers of OA pathogenesis through integrated multi‐omics and functional analyses, and to evaluate whether pharmacological targeting of such drivers could offer a repurposable therapeutic strategy for OA.

## 2 Results

### 2.1 Identification of Macrophage‐Associated Candidate Genes in OA Through Integrated Bioinformatics

To explore genes implicated in OA pathogenesis, we analyzed the dataset GSE236924 from GEO. Following PCA, differential expression analysis between OA and healthy samples revealed a total of 761 DEGs (344 downregulated and 417 upregulated; Figure 1A, Figure S1A and Table S2). GO analysis indicated that the upregulated genes were enriched in terms such as “extracellular structure organization”, “extracellular matrix organization”, “collagen‐containing extracellular matrix”, and “extracellular matrix structural constituent”. Furthermore, KEGG pathway analysis suggested these genes might be involved in the “NF‐kappa B signaling pathway” and “Osteoclast differentiation” (Figure 1B). Gene Set Enrichment Analysis (GSEA) performed on the upregulated genes revealed enhanced activity in both extracellular matrix organization and macrophage activation compared to healthy controls (Figure S1B,C). Collectively, these findings imply that upregulated genes may drive OA progression by disrupting ECM homeostasis, affecting osteoclast differentiation, and linking to METs networks. To further investigate the potential genes involved the progression and onset of OA, the WGCNA analysis was used to screen the Hub genes. We performed WGCNA with a soft‑thresholding power (β) of 5 (Figure S1D), constructing a co‑expression network that identified 20 color‑coded modules (Figure S1E). Module–trait correlations were computed and visualized in a heatmap (Figure 1C), with the MEfirebrick2 module showing the highest correlation to OA. Eight potential Hub genes were subsequently screened based on an MM‐GS correlation cutoff > 0.8 (Figure 1D). Venn demonstrated that five crucial upregulated genes (LSP1, B3GALNT1, MAGED1, NAP1L3, and PCOLCE) might be involved in the pathogenesis of OA (Figure 1E).

**FIGURE 1:**
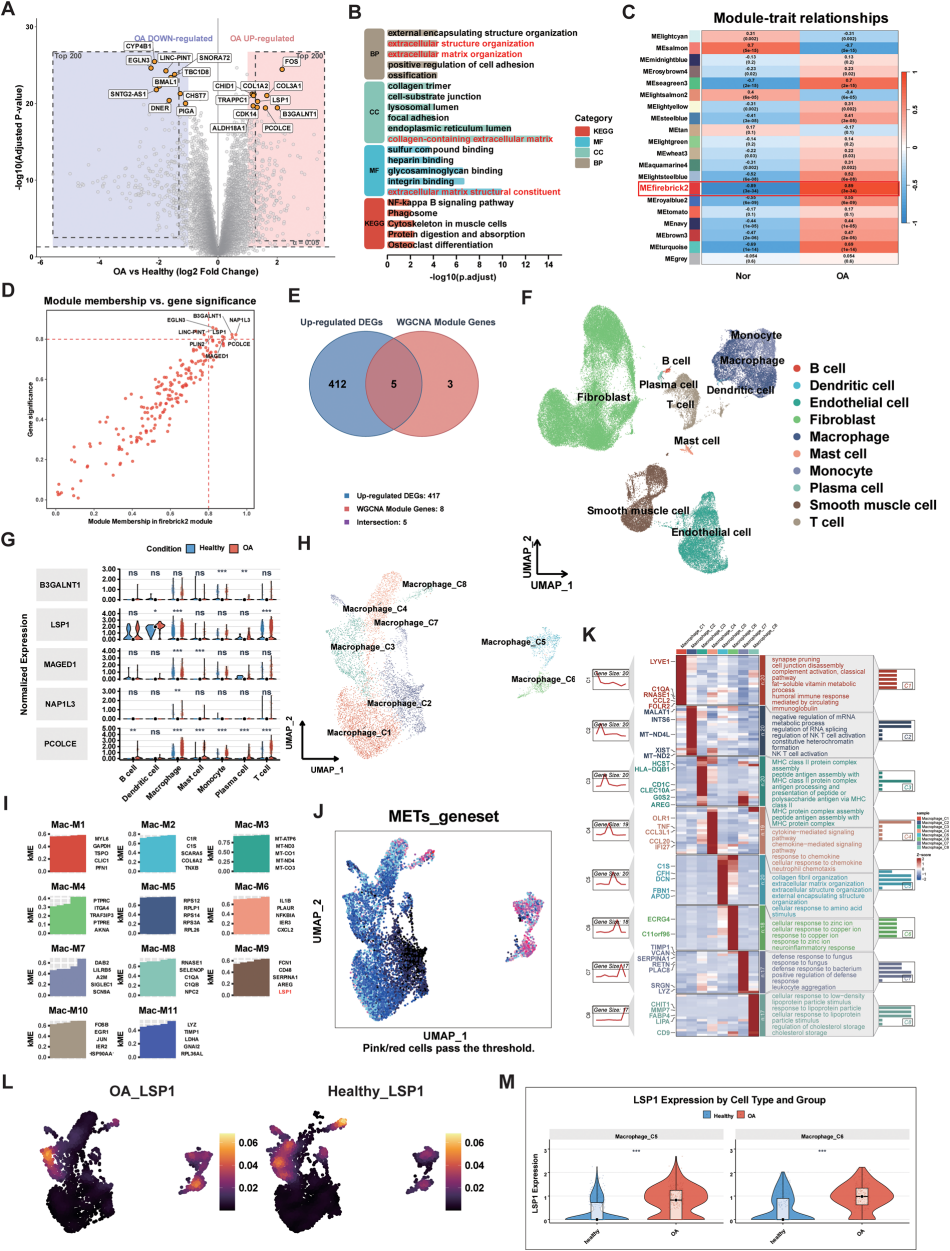
Identification of macrophage‐associated candidate genes in OA through integrated bioinformatics. (A) Volcano plot of differential expression analysis from dataset GSE236924. A total of 417 genes showed ≥2‐fold upregulation. The dashed box highlights the top 200 DEGs ranked by fold change, including LSP1 as one of the most significantly upregulated genes. (B) GO and KEGG enrichment analyses of the 417 upregulated DEGs in OA. The five most significantly enriched pathways were displayed. (C) Heatmap of the correlation between the module and OA, with the firebrick2 module showing a notably high correlation (0.89) with OA. (D) Candidate genes from the firebrick2 module were selected using thresholds of gene significance (GS) > 0.8 and module membership (MM) > 0.8, yielding 8 final candidates. (E) A Venn diagram was used to identify the overlapping genes between the 417 upregulated DEGs in OA patients and the 8 genes selected from the WGCNA firebrick2 module. (F) Single cell landscape of OA in GSE216651 cohort and cells were classified into 10 clusters. (G) Violin plots were used to visualize the expression differences of five key genes across immune cell subsets, in which revealed that four of these genes were significantly upregulated in macrophages from the OA group. (H) UMAP projection of macrophages from single‐cell data, colored by the 8 identified subclusters (Macrophage_C1–C8). (I) hdWGCNA analysis performed on macrophages resulted in the identification of 11 distinct modules. (J) The AUCell algorithm was used to analyze the METs‐related gene set obtained from the GeneCards database. The results showed higher enrichment scores for this gene set in the C5 and C6 subpopulations. (K) Differentially expressed genes across subpopulations were computed using the FindAllMarkers function and visualized in a heatmap. The right side of the heatmap displays the GO enrichment results for the corresponding subpopulation‐specific differentially expressed genes. (L) A density plot was used to compare LSP1 expression levels in macrophage subcluster between the OA and control groups. (M) The expression of LSP1 in C5 and C6 subpopulations from macrophages.

We performed a comprehensive analysis of scRNA sequencing data from OA to delineate the expression patterns of LSP1, B3GALNT1, MAGED1, NAP1L3, and PCOLCE and to assess their potential roles in OA progression (Figure S1G,H). Cells were categorized into 10 major types: B cells, dendritic cells, endothelial cells, fibroblasts, macrophages, mast cells, monocytes, plasma cells, smooth muscle cells, and T cells (Figure 1F). Pseudo‑bulk analysis of the single‑cell data revealed that all five crucial genes were expressed at higher levels in the OA group compared with controls, validating our previous microarray findings (Figure S1F). Furthermore, we applied the COSG algorithm to identify marker genes for each cell population (Figure S1I–K and Table S3). Differentially expressed genes between cell subpopulations were identified with the “FindAllMarkers” function and visualized in a volcano plot (Figure S1L and Table S4). Although multiple innate and adaptive immune cell types have been extensively documented to be involved in OA pathogenesis, their precise roles in OA initiation or progression remain incompletely understood [19]. Visualization of the five key genes across immune cell subsets showed that four (LSP1, MAGED1, NAP1L3, and PCOLCE) were significantly upregulated in OA macrophages, with differences more marked than in other cell types (Figure 1G). This indicates that these genes may promote OA progression, potentially through the modulation of macrophage function and activity.

### 2.2 LSP1 is a Hub Gene Enriched in Distinct Macrophage Subpopulations Associated With Extracellular Matrix and METs

To explore whether LSP1, MAGED1, NAP1L3, and PCOLCE regulate OA pathogenesis via macrophages, we analyzed macrophages from single‐cell data. After extraction, UMAP clustering identified eight subsets (C1–C8) (Figure 1H). Marker genes were defined using COSG (Table S5). hdWGCNA (soft threshold power = 3, Figure S2A) revealed 11 co‐expression modules (M1–M11) (Figure 1I, Figure S2D, Table S6). Among the four genes upregulated in OA macrophages, only LSP1 was identified as a hub gene by hdWGCNA (module M9, kME = 0.531), whereas MAGED1, NAP1L3, and PCOLCE did not meet the hub gene criteria in any macrophage module (Figure S2B and Table S6). M9 was mainly expressed in subsets C2, C3, C5 and C6 (Figure S2C).

Given that LSP1 emerged as the sole hub gene among the candidates, we next examined its known biological functions. LSP1—also known as pp52—is an F‐actin‐binding protein predominantly expressed in hematopoietic cells, including macrophages, where it regulates cytoskeletal remodeling and cell motility [33, 34, 35]. While LSP1 has been implicated in immune cell infiltration in other inflammatory contexts [36, 37], its role in OA pathogenesis had not been previously explored.

We therefore investigated whether LSP1 expression in OA macrophages correlates with METs‐related transcriptional programs. A METs‐related gene set (GeneCards) was subjected to AUCell analysis (threshold = 0.57; 1210 active cells, Figure S2E,F). METs activity was highly enriched in C5 and C6, consistent with high LSP1 expression (Figure 1J). In contrast, M1/M2 marker gene sets showed almost no activity above threshold, indicating that these macrophages do not exhibit classical M1/M2 polarization (Figure S2G,H). Thus, high LSP1 expression correlates with METs rather than M1/M2 states.

GO enrichment analysis of subset‐specific differentially expressed genes (FindAllMarkers) showed that C5 was enriched in extracellular matrix‐related pathways, while C6 was enriched in responses to copper/zinc ions (linked to SOD1) (Figure 1K). Density plots revealed that, relative to controls, LSP1 expression was increased in OA subsets C5 and C6 but decreased in C8 (Figure 1L,M).

Pseudotime BEAM analysis showed two distinct fate branches with low (Fate 1) and high (Fate 2) LSP1 expression (Figure S2I). Cellchat analysis indicated that, compared with healthy controls, OA macrophages had enhanced communication strength in IL‐1, TNF, and CXCL pathways (Figure S2J). Trajectory analysis suggested that C5 and C6 are terminally differentiated states derived from C2, distinct from C1 (Figure S2K). In OA, incoming and outgoing signaling strengths of C5 and C6 were higher than in controls, indicating more active intercellular communication under disease conditions (Figure S2L–N).

Collectively, LSP1 is identified as an OA‐upregulated regulator in macrophage subsets C5/C6 that drives cartilage breakdown via METs—distinct from classical polarization—and is linked to enhanced inflammatory signaling and intercellular communication, positioning LSP1 as a central pathogenic driver in OA.

### 2.3 Functional Validation of LSP1 in Promoting Inflammation, METs Formation, and OA Progression In Vitro and In Vivo

In RAW264.7 macrophages, Lsp1 knockdown (si‐Lsp1; confirmed by RT‐qPCR and Western blot, Figure S3A,B) significantly suppressed LPS‐induced upregulation of *Il6*, *Tnfα*, and *Cxcl10* relative to control cells (Figure 2A). Knockdown of Lsp1 also reduced LPS‐elevated cell‐free DNA (cfDNA) levels in the supernatant, whereas unstimulated groups exhibited no significant change (Figure 2B). Moreover, Western blot analysis demonstrated that Lsp1 knockdown attenuated the LPS‐induced expression of CitH3, a marker of METs, at multiple time points (Figure 2C; Figure S3C,D). Conversely, Lsp1 overexpression (Figure S4A,B) enhanced CitH3 expression following LPS stimulation (Figure S4C) and promoted LPS‐induced cfDNA accumulation (Figure S4D). Collectively, these findings indicate that LSP1 facilitates METs formation and release.

**FIGURE 2:**
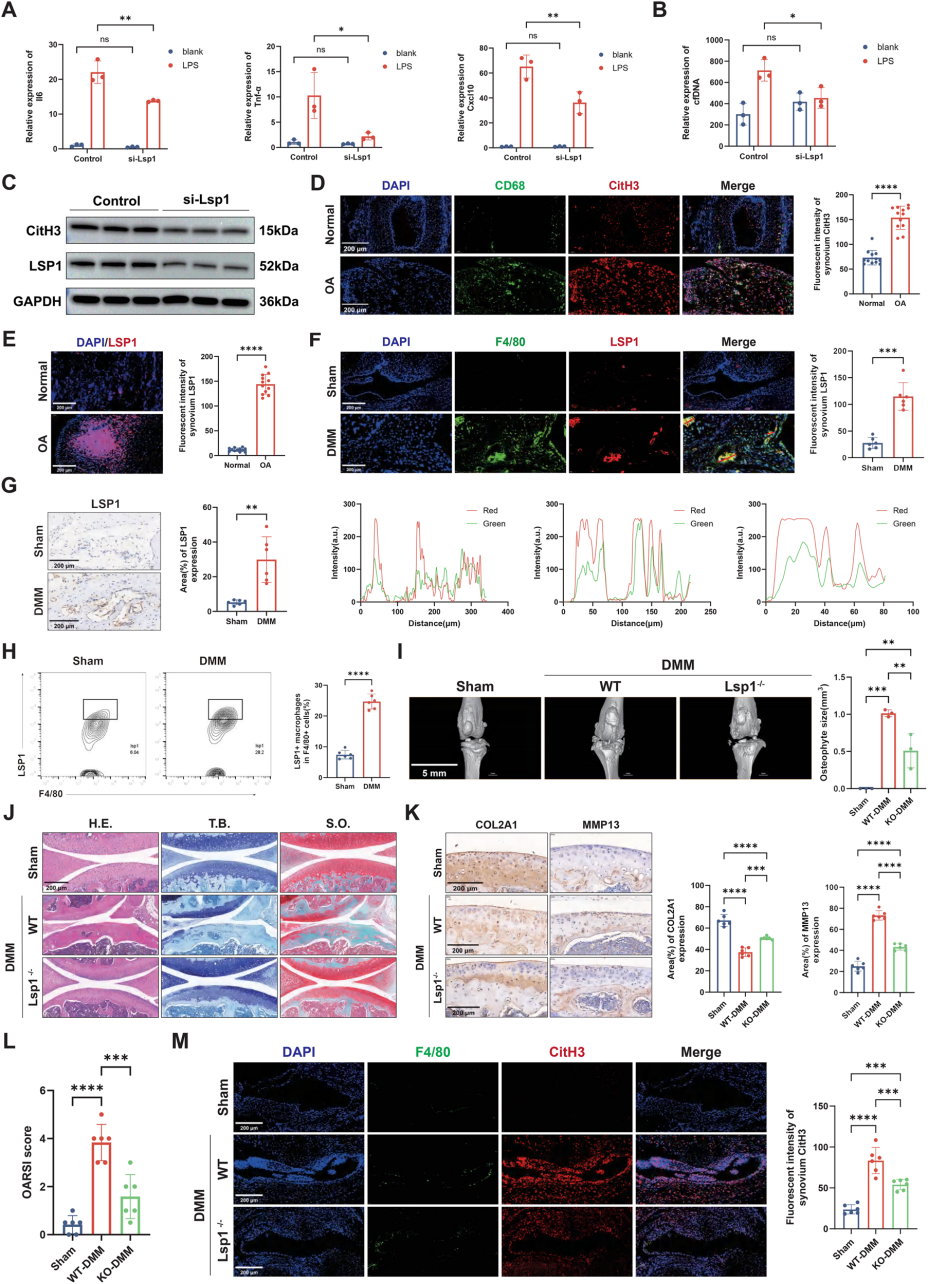
Functional validation of LSP1 in promoting inflammation, METs formation, and OA progression in vitro and in vivo. (A) The relative mRNA levels of Il6, Tnfα, and Cxcl10 were analyzed by RT‐qPCR. (B) The cfDNA levels in the cell supernatant from different groups were measured by ELISA. (C) Western blot analysis of CitH3 expression in control and si‐Lsp1 cells following LPS treatment over 0, 6, 12, and 24 h. (D) Immunofluorescence was performed to examine the expression of CD68 (green) and CitH3 (red) in the knee synovium of patients with OA (n = 12) and that of control subjects (n = 12), scale bar: 200 µm. (E) Immunofluorescence was performed to examine the expression of LSP1 (red) in the knee synovium of patients with OA(n = 12) and that of control subjects (n = 12), scale bar: 200 µm. (F) Immunofluorescence was performed to examine the expression of F4/80 (green) and LSP1 (red) in the knee synovium of DMM‐induced OA model mice (n = 6) and sham‐operated controls (n = 6), scale bar: 200 µm. (G) Immunohistochemistry was performed to examine LSP1 expression in the synovium of DMM‐induced OA model mice (n = 6) and sham‐operated controls (n = 6), scale bar: 200 µm. (H) Flow cytometry was employed to detect the proportion of LSP1^+^ macrophages among total macrophages in the synovial tissues of mice in the model group (n = 6) versus the sham‐operated group (n = 6). (I) Micro‐CT analysis of knee joints from Sham (n = 6), DMM‐WT (n = 6), and DMM‐Lsp1^−/−^ (n = 6) mice showed that osteophyte volume in DMM‐Lsp1^−/−^ mice was significantly smaller than that in DMM‐WT mice. scale bar: 5 mm. (J) Representative H.E., T.B., and S.O. staining images of cartilage tissues from Sham (n = 6), DMM‐WT (n = 6), and DMM‐Lsp1^−/−^ (n = 6) mice, scale bar: 200 µm. (K) Immunohistochemistry was performed to analyze COL2A1 and MMP13 expression in the cartilage of Sham (n = 6), DMM‐WT (n = 6), and DMM‐Lsp1^−/−^ (n = 6) mice, scale bar: 200 µm. (L) OA severity in each group of mice was assessed using the OARSI scoring system based on S.O. staining results. (M) Immunofluorescence was performed to assay the expression of F4/80 (green) and CitH3 (red) in the knee synovium of Sham (n = 6), DMM‐WT (n = 6), and DMM‐Lsp1^−/−^ (n = 6) mice, scale bar: 200 µm.

In human synovial tissues obtained from OA patients (n = 12) and control individuals (n = 12), immunofluorescence staining revealed upregulated CitH3 expression in OA macrophages (Figure 2D), and LSP1 expression was significantly higher in OA samples compared with controls (Figure 2E). Consistently, synovial LSP1 levels were elevated in DMM‐induced OA mice relative to sham‐operated controls (Figure 2F,G). Flow cytometric analysis further demonstrated an increased proportion of LSP1^+^ macrophages among total synovial macrophages in DMM mice versus sham mice (Figure S4E; Figure 2H).

To investigate the in vivo function of LSP1, we generated Lsp1 knockout mice using CRISPR‐Cas9 (genotyping, Figure S4F). Following DMM surgery, Lsp1^−^/^−^ mice developed significantly smaller osteophytes than wild‐type (WT) DMM mice (Figure 2I). Histological evaluation with H.E., Toluidine Blue, and Safranin O staining revealed less severe cartilage damage in Lsp1^−^/^−^‐DMM mice (Figure 2J). Furthermore, cartilage from Lsp1^−^/^−^‐DMM mice exhibited elevated COL2A1 expression and reduced MMP13 positivity (Figure 2K), along with lower OARSI histological scores (Figure 2L). Immunofluorescence additionally showed diminished expression of the macrophage marker F4/80 and the METs marker CitH3 in the cartilage of Lsp1^−^/^−^‐DMM mice (Figure 2M), indicating decreased local macrophage infiltration and METs formation. Collectively, these data establish that LSP1 drives OA progression by promoting macrophage inflammation and METs formation, positioning LSP1 as a promising therapeutic target.

### 2.4 BMDM Assays and Bone Marrow Reconstitution Validate Macrophage‐Derived LSP1 in METs and OA Progression

Immunofluorescence staining verified the complete ablation of LSP1 expression in global Lsp1^−/−^ mice (Figure 3A). However, this conventional knockout strategy precludes discrimination between the contributions of macrophage‐expressed LSP1 and LSP1 derived from other cell types to OA pathogenesis. To resolve this ambiguity, bone marrow‐derived macrophages (BMDMs) were isolated from wild‐type (WT) and Lsp1^−/−^ mice to examine the role of LSP1 in METs formation. Following LPS challenge, the level of CitH3 was significantly diminished in Lsp1^−/−^ BMDMs relative to WT controls, indicating impaired METs assembly (Figure 3B). In line with this observation, we next assessed the molecular status of chondrocytes in the co‐culture system by Western blot. Chondrocytes co‐cultured with LPS‐primed Lsp1^−/−^ BMDMs exhibited significantly higher COL2A1 expression and lower MMP3 and MMP13 levels compared with those co‐cultured with WT BMDMs (Figure 3C), indicating that LSP1 deficiency in macrophages preserves chondrocyte matrix integrity at the molecular level.

**FIGURE 3:**
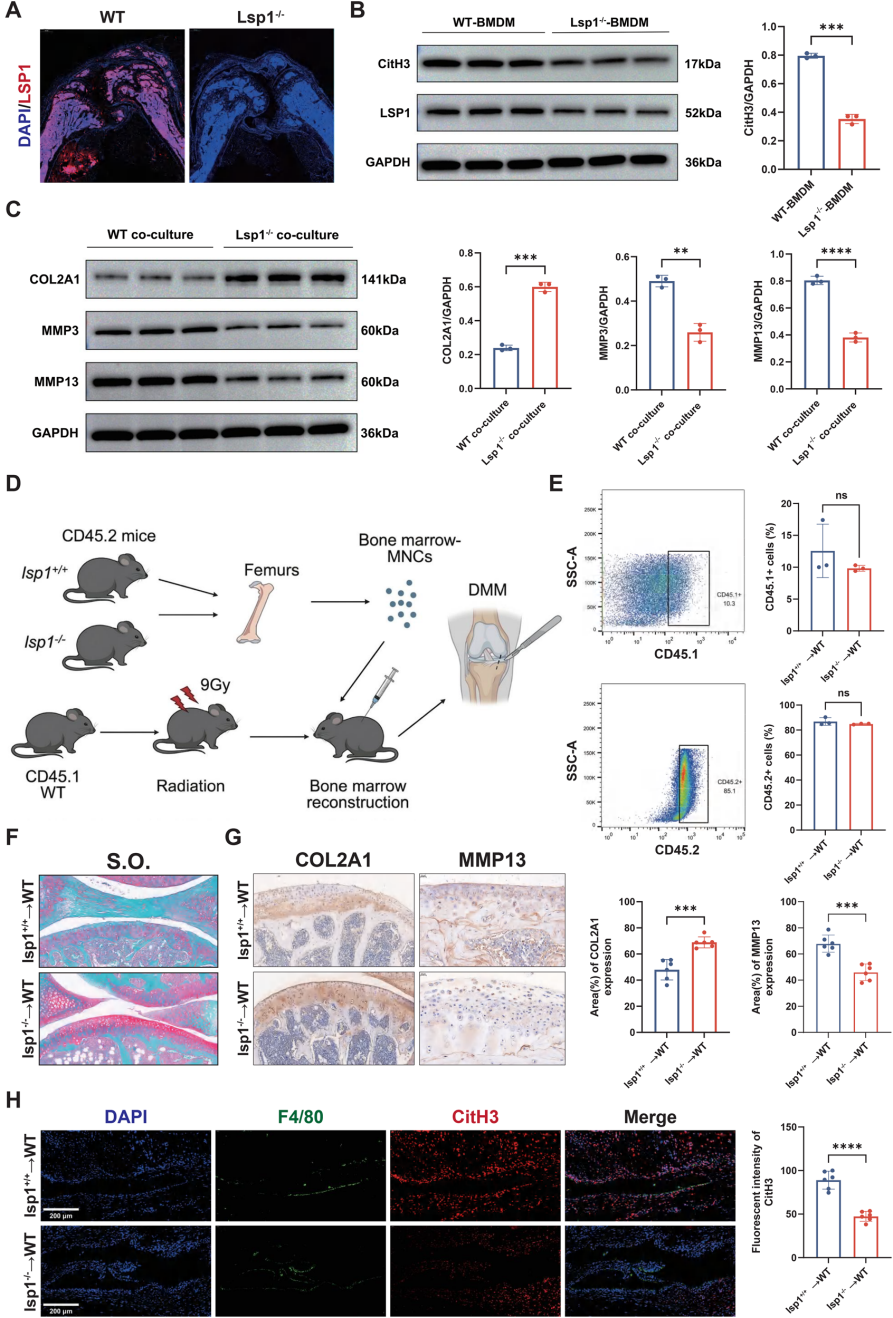
Macrophage‐derived LSP1 functions in OA verified by bone marrow reconstitution. (A) Immunofluorescence assessment of Lsp1 gene knockout efficiency in murine knee joint tissues. (B) The Western blot was used to detect the expression of CitH3 in BMDMs from WT and Lsp1^−/−^ mice after LPS stimulation. (C) Western blot analysis was performed to assess the changes in cartilage‐related protein expression in primary chondrocytes co‐cultured with LPS‐prestimulated BMDMs from WT or Lsp1^−/−^ mice. (D) Schematic of bone marrow reconstitution assay: Bone marrow MNCs from CD45.2 Lsp1^+/+^/Lsp1^−/−^ mice were transplanted into 9Gy‐irradiated CD45.1 WT mice, followed by DMM surgery. (E) Flow cytometric analysis of synovial digests from reconstituted mice (Lsp1^+^/^+^→WT and Lsp1^−^/^−^→WT) at 8 weeks post‐transplantation. Left: representative plots showing CD45.1 (recipient‐derived) and CD45.2 (donor‐derived) cells (SSC‐A vs. CD45.1/CD45.2). Right: quantification of donor‐derived (CD45.2^+^) cell percentages. (F) Representative S.O. staining images from Lsp1^+/+^→WT and Lsp1^−/−^→WT mice. (G) IHC analysis of COL2A1 and MMP13 in joint tissues of reconstituted DMM mice; quantitative analysis of positive expression area (%) in Lsp1^+/+^→WT and Lsp1^−/−^→WT groups. (H) IF staining of DAPI/F4/80/CitH3 in reconstituted joint tissues with merged images; quantitative analysis of CitH3 fluorescent intensity in Lsp1^+/+^→WT and Lsp1^−/−^→WT groups.

To further define the in vivo contribution of macrophage‐specific LSP1 to OA development, we generated Lsp1^+/+^→WT and Lsp1^−/−^→WT bone marrow chimeric mice via lethal irradiation (9 Gy) and subsequent bone marrow mononuclear cell (BM‐MNC) transplantation. After stable hematopoietic reconstitution, all mice were subjected to DMM surgery (Figure 3D). To assess the efficiency of hematopoietic reconstitution and confirm comparable donor chimerism between experimental groups, flow cytometry was performed on synovial digests at 8 weeks post‐transplantation using CD45.1/CD45.2 congenic markers. As shown in Figure 3E, donor‐derived (CD45.2^+^) cells comprised over 80% of total synovial cells in both Lsp1^+^/^+^→WT and Lsp1^−^/^−^→WT chimeras, whereas recipient‐derived (CD45.1^+^) cells accounted for approximately 10%. Importantly, the percentage of donor chimerism did not differ significantly between the two groups (n  =  3 per group). These results demonstrate that lethal irradiation and bone marrow transplantation successfully reconstituted the synovial myeloid compartment with donor‐derived cells, and that Lsp1 deletion does not affect the engraftment efficiency or overall cellularity. Consequently, any subsequent differences in cartilage pathology or METs‐related markers between the two chimeric groups can be specifically attributed to the absence of Lsp1 in donor‐derived macrophages rather than to confounding variations in chimerism levels.

Lsp1^+/+^→WT chimeras displayed exacerbated OA cartilage lesions, characterized by more severe cartilage damage as shown by S.O. staining (Figure 3F), reduced COL2A1 abundance, elevated MMP13 expression (Figure 3G), and increased CitH3 fluorescence intensity (Figure 3H). Collectively, these data establish that macrophage‐derived LSP1 deficiency restrains METs formation and attenuates OA progression in vivo.

### 2.5 LSP1 Promotes METs Formation via NOX‐Dependent and mtROS Pathways, Exacerbating Chondrocyte Damage

To determine whether LSP1‐mediated METs directly inflict damage upon chondrocytes, conditioned medium containing METs derived from LPS‐stimulated macrophages was transferred to chondrocyte cultures. Relative to control medium, this treatment led to a marked reduction in COL2A1 expression and concomitant upregulation of MMP3 and MMP13 (Figure 4A). In a Transwell co‐culture system with macrophages seeded in the upper chamber and chondrocytes in the lower chamber, LPS‐induced chondrocyte injury was significantly attenuated by either DNase I (which degrades pre‐formed METs) or a peptidylarginine deiminase inhibitor (PADi, which blocks METs formation). These protective effects were evidenced by restoration of COL2A1 expression and suppression of MMP3 and MMP13 levels (Figure 4B–D). Collectively, these findings establish that METs directly contribute to chondrocyte injury.

**FIGURE 4:**
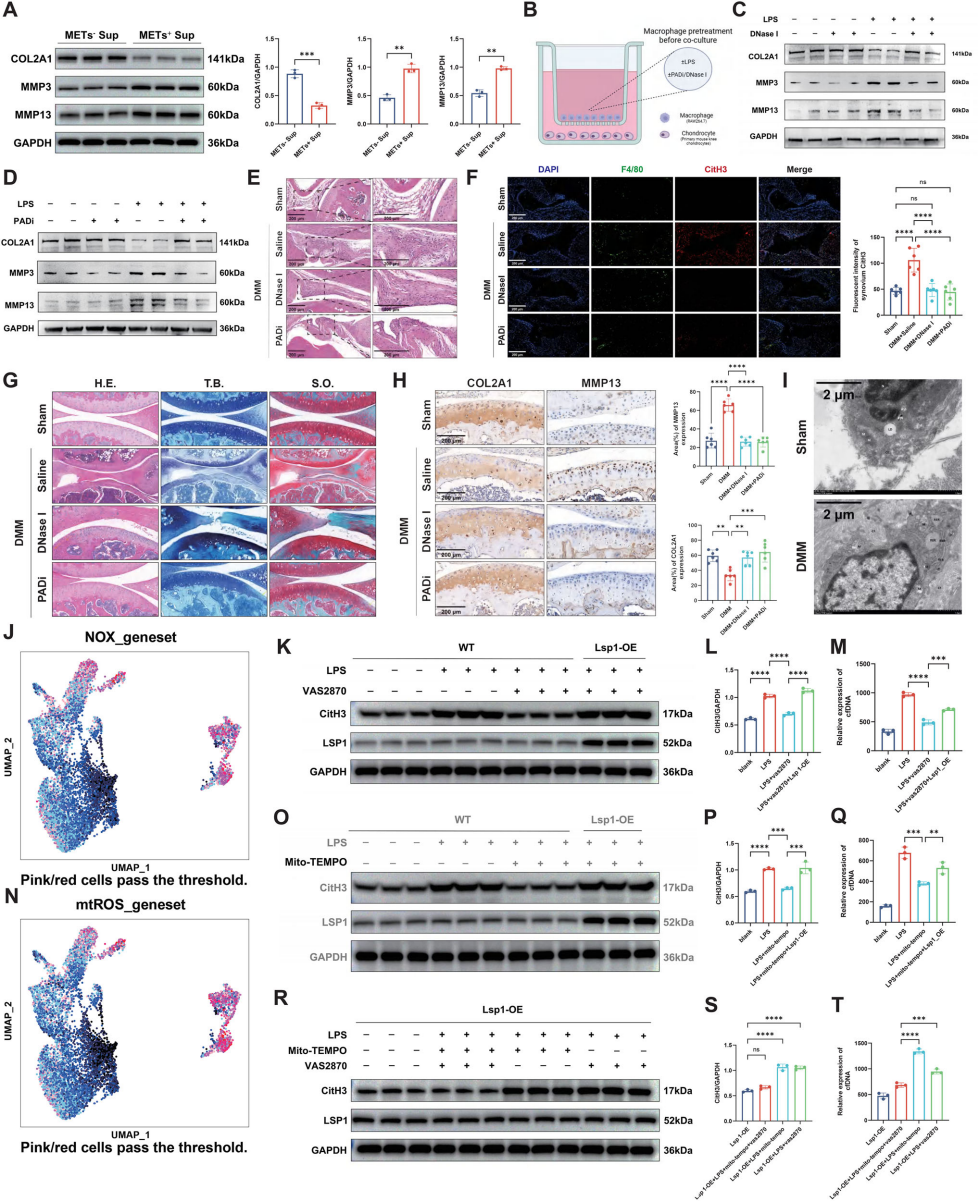
LSP1 promotes METs formation via NOX‐dependent and mtROS pathways, exacerbating chondrocyte damage. (A) The Western blot was used to analyze the expression of COL2A1, MMP13, and MMP3 in chondrocyte with or without METs. (B) A coculture system of RAW264.7 cells and chondrocytes were established using a Transwell setup, with RAW264.7 cells seeded in the upper chamber and chondrocytes in the lower chamber. The system was treated with specified concentrations of LPS (to stimulate RAW264.7 cells), DNase I (to clear METs), and a PAD4 inhibitor (PADi, to inhibit METs formation). (C) Western blot analysis of COL2A1, MMP13, and MMP3 expression in chondrocytes co‐cultured with LPS‐stimulated RAW264.7 macrophages and treated with DNase I. (D) Western blot analysis of COL2A1, MMP13, and MMP3 expression in chondrocytes co‐cultured with LPS‐stimulated RAW264.7 macrophages and treated with PADi. (E) Representative H.E. staining images of synovial tissues from each group, scale bar: 200 µm. (F) Immunofluorescence was performed to show the expression of F4/80 (green) and CitH3 (red) in the knee synovium of Sham (n = 6), DMM‐Saline (n = 6), DMM‐DNase I (n = 6), and DMM‐PADi mice, scale bar: 200 µm. (G) Representative H.E., T.B., and S.O. staining images of cartilage tissues from Sham (n = 6), DMM‐Saline (n = 6), DMM‐DNase I (n = 6), and DMM‐PADi mice, scale bar: 200 µm. (H) Immunohistochemistry was performed to examine COL2A1 and MMP13 expression in the cartilage of Sham (n = 6), DMM‐Saline (n = 6), DMM‐DNase I (n = 6), and DMM‐PADi mice, scale bar: 200 µm. (I) Transmission electron microscopy was performed to examine mitochondrial ultrastructural changes in macrophages within the synovial tissues of DMM‐induced OA model mice compared with control mice. (J) The AUCell algorithm was used to analyze the NOX‐related gene set obtained from the GeneCards database. (K) Subsequent panels K–T used the following experimental design: RAW264.7 macrophages were transfected with Lsp1‐overexpressing plasmids (or vector controls) and then stimulated with LPS (100 ng/mL) for the indicated durations. Cells were treated with VAS2870 (NOX inhibitor), Mito‐TEMPO (mtROS inhibitor), or both, as indicated, prior to analysis of CitH3 expression and cfDNA release. Western blot analysis of CitH3 expression in RAW264.7 treated with LPS and the NOX inhibitor (VAS2870), or overexpressing Lsp1 (Lsp1‐OE). (L) The statistical graph showed the expression levels of CitH3 in each group of cells. (M) The cfDNA levels in the supernatant of RAW264.7 treated with LPS and the NOX inhibitor (VAS2870), or Lsp1‐OE were detected by ELISA. (N) The AUCell algorithm was used to analyze the mtROS‐related gene set obtained from the GeneCards database. (O) Western blot analysis of CitH3 expression in RAW264.7 treated with LPS and the mtROS‐inhibitor (Mito‐TEMPO), compared to those Lsp1‐OE. (P) The statistical graph showed the expression levels of CitH3 in each group of cells. (Q) ELISA quantification of cfDNA concentration in the supernatant of RAW264.7 treated with LPS and Mito‐TEMPO, or Lsp1‐OE. (R) Western blot analysis of CitH3 expression in Lsp1‐overexpressing RAW264.7 treated with VAS2870, Mito‐TEMPO, or VAS2870 + Mito‐TEMPO. (S) The statistical graph showed the expression levels of CitH3 in each group of cells. (T) ELISA quantification of cfDNA concentration in the supernatant of Lsp1‐overexpressing RAW264.7 treated with VAS2870, Mito‐TEMPO, or VAS2870 + Mito‐TEMPO.

In the DMM‐induced murine model of OA, intra‐articular administration of DNase I or PADi markedly ameliorated synovial inflammation, as assessed by H&E staining (Figure 4E). Immunofluorescence analyses further revealed diminished expression of the macrophage marker F4/80 and the METs marker CitH3 within cartilage (Figure 4F). Histological evaluation (H&E, Toluidine Blue, and Safranin O) demonstrated that METs clearance substantially mitigated structural cartilage deterioration (Figure 4G), while immunohistochemistry showed increased COL2A1 and decreased MMP13 levels in cartilage (Figure 4H). These results collectively indicate that local elimination of METs alleviates OA progression.

Given the well‐established similarity between METs and neutrophil extracellular traps (NETs), we hypothesized that METs formation similarly relies on enhanced NOX activity and mitochondrial reactive oxygen species (mtROS) accumulation. Synovial macrophages from OA mice exhibited pronounced mitochondrial ultrastructural abnormalities—including swelling, matrix pallor, and cristae fragmentation—compared to the normal morphology observed in sham controls (Figure 4I). AUCell analysis revealed that NOX‐related gene sets were significantly enriched in cell subsets largely overlapping with LSP1‐high populations, suggesting a role for NOX in LSP1‐mediated METs formation (Figure 4J). To experimentally validate this, RAW264.7 macrophages were transfected with Lsp1‐overexpressing plasmids (or empty vector controls) and then stimulated with LPS (100 ng/mL) prior to inhibitor treatment. In Lsp1‐overexpressing cells, the NOX inhibitor VAS2870 suppressed LSP1‐induced CitH3 expression (Figure 4K,L) and cfDNA accumulation (Figure 4M). Similarly, mtROS‐related gene sets showed overlapping enrichment with LSP1‐high subsets (Figure 4N), and the mtROS inhibitor Mito‐TEMPO attenuated LSP1‐driven CitH3 expression and cfDNA accumulation (Figure 4O–Q). To delineate the interplay between NOX and mtROS pathways, we applied their respective inhibitors individually or in combination under Lsp1 overexpression conditions. While single‐pathway inhibition partially reduced CitH3 levels—though they remained above unstimulated controls—dual blockade resulted in a more substantial decrease in both CitH3 and cfDNA, demonstrating superior efficacy over either inhibitor alone (Figure 4R–T). These data indicate that LSP1‐mediated METs formation, akin to NOX‐dependent NETosis, requires concurrent signaling through both NOX and mtROS pathways. Importantly, this mechanistic paradigm was recapitulated in human THP‐1 macrophages (Figure S5A–F).

### 2.6 LSP1 Exacerbates Oxidative Stress and OA Progression by Binding to and Inhibiting SOD1

To elucidate how LSP1 regulates the NOX and mtROS pathways, we first employed IP‐MS and identified superoxide dismutase 1 (SOD1) as a potential binding partner of LSP1 (Table S9). Among the top 50 proteins identified, we excluded antibody contaminants and abundant structural proteins (e.g., actins, tubulins, keratins, myosin, and vimentin), which are common background in IP‐MS experiments. After this filtering, SOD1 emerged as the only candidate with a direct enzymatic function in superoxide anion scavenging and a plausible mechanistic link to the redox imbalance underlying NOX and mtROS activation. We therefore prioritized SOD1 for subsequent validation. Accordingly, we identified SOD1 as a potential binding partner of LSP1 (Figure 5A and Table S9). SOD1 effectively scavenges excess intracellular superoxide anions, maintains mitochondrial homeostasis, and alleviates oxidative damage. Co‐IP confirmed the interaction between LSP1 and SOD1 (Figure 5B), and SPR analysis further demonstrated their direct binding (Figure 5C). Immunofluorescence co‐localisation showed overlapping spatial distribution of LSP1 and SOD1, with signals detected in both the cytosol and the nucleus (Figure 5D). The predominant LSP1‐SOD1 co‐localization was cytosolic, consistent with their proposed role in regulating cytoplasmic superoxide levels. This cytosolic co‐localisation supports their direct interaction in the cytoplasmic compartment and suggests that LSP1 may directly bind and inhibit SOD1 activity, thereby impairing cellular superoxide‐scavenging capacity and ultimately exacerbating oxidative stress in synovial macrophages.

**FIGURE 5:**
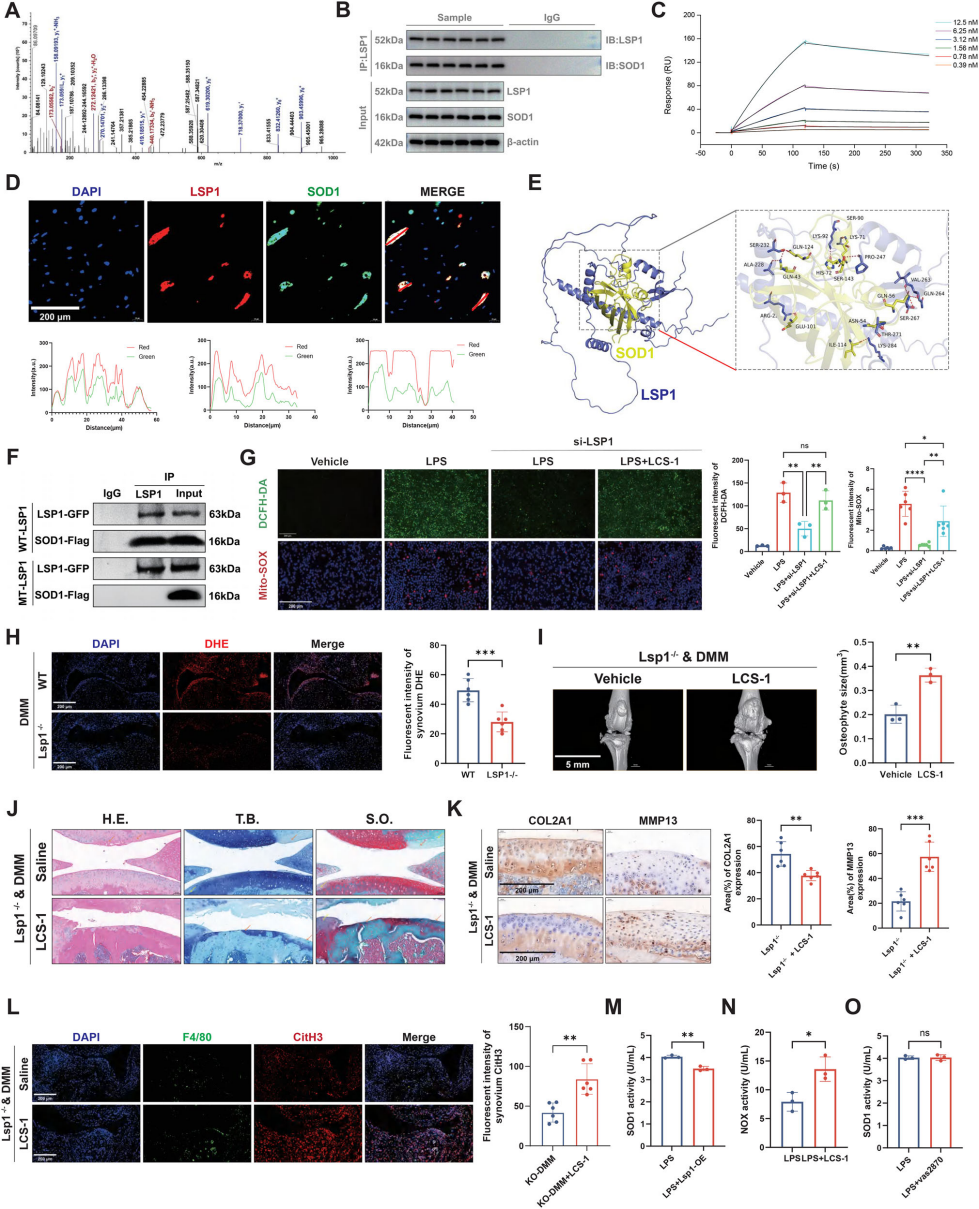
LSP1 exacerbates oxidative stress and OA progression by binding to and inhibiting SOD1. (A) Schematic of the IP‐MS experiment identifying the antioxidant enzyme SOD1 as a binding partner of LSP1. (B) Co‐immunoprecipitation (Co‐IP) of LSP1 in RAW264.7. Immunoblots were probed with anti‐LSP1 and anti‐SOD1. n = 6. (C) Affinity sensing diagram and fitting curve of a series of concentrations of SOD1 compounds with LSP1. The interaction was detected with a Biacore T200 using CM5 chips and assessed using Biacore T200 Evaluation Software 3.0. (D) Immunofluorescence analysis showing co‐localization of LSP1 and SOD1. (E) Molecular docking model of the LSP1‐SOD1 interaction, highlighting key hydrogen bonds formed by LSP1 residues 221 and 232. (F) Co‐IP analysis demonstrated that truncation of the LSP1 domain containing residues 221 and 232 abolishes its interaction with SOD1. (G) Representative DCFH‐DA (2′,7′‐dichlorodihydrofluorescein diacetate) and Mito‐SOX staining images of cells treated with LPS or LPS+LCS‐1. Scale bar: 200 µm. (H) ROS levels in the knee synovium were assessed by Dihydroethidium (DHE) staining (red) in DMM‑WT (n = 6) and DMM‑Lsp1^−/−^ (n = 6) mice. Scale bar: 200 µm. (I) Representative micro‐CT images of knee joints from vehicle‐ or LCS‐1‐treated DMM‐Lsp1^−/−^ mice. scale bar: 5 mm. (J) Representative H.E., T.B., and S.O. staining images of cartilage tissues from DMM‐Lsp1^−/−^ mice with or without LCS‐1 treatment, scale bar: 200 µm. (K) Immunohistochemistry was performed to examine COL2A1 and MMP13 expression in the cartilage of DMM‐Lsp1^−/−^ (n = 6) and DMM‐Lsp1^−/−^+LCS‐1 (n = 6) mice, scale bar: 200 µm. (L) Immunofluorescence was performed to show the expression of F4/80 (green) and CitH3 (red) in the knee synovium from vehicle‐ or LCS‐1‐treated DMM‐Lsp1^−/−^ mice, scale bar: 200 µm. (M) The relative SOD1 levels were analyzed in Lsp1‐overexpressing RAW264.7 treated with LPS. (N) The relative NOX levels were analyzed in RAW264.7 treated with LPS or LPS & LCS‐1. (O) The relative SOD1 levels were analyzed in RAW264.7 treated with LPS or LPS & VAS2870.

To clarify the binding mode, we performed molecular docking, which predicted multiple potential interaction sites; among these, residues 221 and 232 of LSP1 were predicted to form strong hydrogen bonds (Figure 5E). Point mutations at these sites did not significantly weaken the interaction, but truncation of the domain containing both residues abolished SOD1 binding (Figure 5F), indicating that this region is critical for the interaction. LPS stimulation triggered oxidative stress in macrophages, as evidenced by elevated ROS and mtROS levels; Lsp1 knockdown restored SOD1 activity and reduced ROS and mtROS levels. Adding the SOD1 inhibitor LCS‐1 under these conditions again induced ROS and mtROS accumulation (Figure 5G), confirming that LSP1 inhibits SOD1 activity. Of note, the LSP1–SOD1 interaction is defined by direct protein–protein binding, and the rescue of ROS by LCS‐1 reflects inhibition of SOD1 enzymatic activity rather than restoration of the LSP1–SOD1 complex. Consistent with this, ROS scavengers such as Mito‐TEMPO and NAC would not be expected to modulate the LSP1/SOD1 complex, as they act downstream of SOD1 activity. Furthermore, Lsp1 knockout markedly lowered ROS levels in synovial tissue of DMM‐induced OA mice (Figure 5H). Intra‐articular injection of LCS‐1 into Lsp1‐knockout OA mice increased osteophyte volume (Figure 5I), exacerbated cartilage damage (Figure 5J), decreased COL2A1 and increased MMP13 expression in cartilage (Figure 5K), and elevated synovial F4/80 and CitH3 levels (Figure 5L), indicating that inhibiting SOD1 promotes METs formation. Furthermore, Lsp1 overexpression suppressed the LPS‐induced increase in SOD1 enzymatic activity (Figure 5M), while LCS‐1 treatment subsequently elevated NOX activity (Figure 5N), suggesting that SOD1 inhibition further activates the NOX pathway to drive METs production. To further delineate the directionality between SOD1 inhibition and NOX activation, we examined whether NOX inhibition could reciprocally affect SOD1 activity. As shown in Figure 5O, treatment with the NOX inhibitor VAS2870 did not significantly alter SOD1 enzymatic activity in LPS‐stimulated macrophages compared with LPS alone, indicating that NOX lies downstream of SOD1 in this regulatory axis. The results in Figure 5N,O support a unidirectional relationship in which LSP1‐mediated SOD1 inhibition acts upstream of NOX activation, rather than operating in a mutually reinforcing loop. Collectively, these findings establish that LSP1 binds and inhibits SOD1, impairing superoxide clearance. This redox imbalance co‐activates NOX and mtROS pathways to drive METs release from macrophages, thereby exacerbating cartilage degradation and OA progression.

### 2.7 The Cardiac Glycoside Digoxin Alleviates OA by Targeting the LSP1‐SOD1 Interaction

To screen for potential drugs targeting LSP1 for OA treatment, we performed a virtual screening based on the key binding domain of LSP1 and identified digoxin as a potential competitive binder (Table S10). Digoxin is an FDA‐approved cardiotonic steroid used for heart failure. It has shown immunomodulatory properties and was reported to alleviate OA‐related pain, potentially through inhibition of synovial macrophage M1‐like polarization [38, 39, 40, 41]. SPR confirmed a high binding affinity between digoxin and LSP1 (Figure 6A). Adding digoxin to the LSP1‑SOD1 binding system significantly inhibited their interaction (Figure 6B), suggesting that digoxin may exert therapeutic effects by blocking LSP1‑SOD1 binding and restoring SOD1 activity.

**FIGURE 6:**
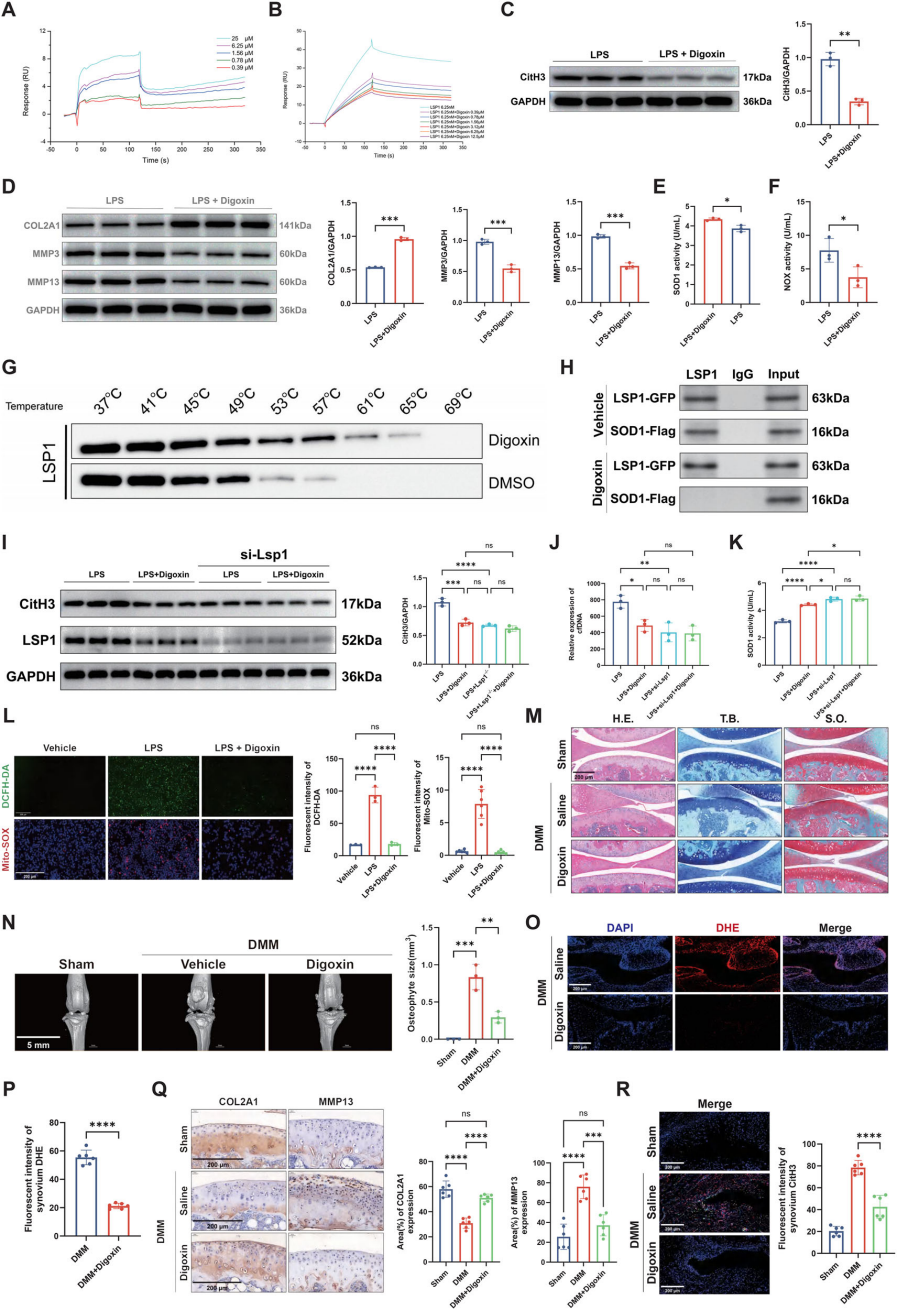
The cardiac glycoside digoxin alleviates OA by targeting the LSP1‐SOD1 interaction. (A) Virtual screening identifies digoxin as a candidate LSP1 binder, validated by surface plasmon resonance. (B) The results of surface plasmon resonance showed that digoxin competitively inhibits the LSP1‐SOD1 protein interaction. (C) Western blot analysis of CitH3 expression in RAW264.7 treated with LPS and LPS+digoxin groups. (D) Western blot analysis of COL2A1, MMP13, and MMP3 expression in chondrocytes co‐cultured with LPS‐stimulated RAW264.7 macrophages and treated with digoxin. (E) The relative SOD1 levels were analyzed in different groups. (F) The relative NOX levels were analyzed in different groups. (G) Cellular thermal shift assay (CETSA) validating that digoxin directly binds to LSP1 in HEK293T cells. (H) Co‐immunoprecipitation (Co‐IP) analysis showing that digoxin treatment disrupts the LSP1‐SOD1 interaction. (I) Western blot analysis of CitH3 expression in RAW264.7 macrophages treated with LPS, LPS + digoxin, LPS + si‐Lsp1 and LPS + si‐Lsp1 + digoxin groups. (J) The cfDNA levels in the cell supernatant from different groups were measured by ELISA. (K) The relative SOD1 levels were analyzed in RAW264.7 from different groups. (L) Representative DCFH‐DA and Mito‐SOX staining images of cells treated with LPS or LPS+digoxin. Scale bar: 200 µm. (M) Representative H.E., T.B., and S.O. staining images of cartilage tissues from sham (n = 6), DMM‐saline (n = 6), and DMM‐digoxin (n = 6) mice, scale bar: 200 µm. (N) Representative micro‐CT images of knee joints from vehicle‐ or digoxin‐treated DMM mice. scale bar: 5 mm. (O) ROS levels in the knee synovium were detected by DHE staining (red) in DMM‐saline (n = 6) and DMM‐digoxin (n = 6) mice. Scale bar: 200 µm. (P) Quantification of DHE fluorescence intensity in synovium from each group. (Q) Immunohistochemistry was performed to examine COL2A1 and MMP13 expression in the cartilage of Sham (n = 6), DMM‐saline (n = 6), and DMM‐digoxin (n = 6) mice, scale bar: 200 µm. (R) Immunofluorescence was performed to show the expression of CitH3 (red) in the knee synovium from Sham (n = 6), DMM‐saline (n = 6), and DMM‐digoxin (n = 6) mice, scale bar: 200 µm.

Previous clinical analyses of the IQVIA database involving 28 397 digoxin users and an equal number of non‐users demonstrated a reduced cumulative incidence of knee or hip OA‐related joint replacement in the digoxin group, suggesting its therapeutic potential in OA [42]. Following oral administration, digoxin enters the systemic circulation and distributes throughout vascularized tissues. Given that the synovium is richly perfused, orally administered digoxin is likely accessible to synovial tissues. The potential contribution of orally administered digoxin to synovial tissues should therefore not be dismissed. While the original study attributed the reduced joint replacement incidence primarily to LRP4‐mediated chondroprotection, our findings raise the possibility that LSP1‐mediated synovial anti‐inflammatory effects represent an additional clinically relevant mechanism. Accordingly, the clinical benefits observed in digoxin users may reflect, at least in part, suppression of synovial inflammation, complementing the previously reported chondroprotective actions. In LPS‐stimulated RAW264.7 cells, titrating digoxin concentrations revealed that 100 nm significantly suppressed CitH3 expression (Figure 6C and Figure S6A–C), indicating inhibition of METs formation, while higher concentrations did not further reduce METs levels. Notably, 100 nm digoxin has previously been reported to alleviate OA by targeting low‐density lipoprotein receptor‐related protein 4 in cartilage, and in vivo studies confirmed that systemic administration at this concentration did not induce cardiac, hepatic, or splenic toxicity in mice [42]. In the Transwell co‑culture system, digoxin alleviated LPS‑induced chondrocyte damage, shown by upregulated COL2A1 and downregulated MMP3 and MMP13 expression (Figure 6D). To determine whether digoxin exerts a comparable inhibitory effect on METs formation in human cells, we conducted experiments in the THP‐1 monocytic cell line and confirmed the efficacy of digoxin in human‐derived cells (Figure S6D). Given that histone H3 phosphorylation may influence its subsequent citrullination, we examined whether digoxin modulates p‐H3 levels to exclude potential crosstalk between these modifications. As shown in Figure S6E, digoxin treatment did not significantly alter p‐H3 levels, indicating that the suppression of CitH3 by digoxin is not mediated through modulation of H3 phosphorylation. Digoxin also increased SOD1 enzymatic activity (Figure 6E) and inhibited the LPS‑induced rise in NOX activity (Figure 6F), suggesting it maintains redox balance to inhibit METs generation.

To further validate the interaction between digoxin and LSP1 in a cellular context, we performed cellular thermal shift assay (CETSA) and co‐immunoprecipitation (Co‐IP) in HEK293T cells. CETSA confirmed that digoxin directly bound to LSP1, as evidenced by increased thermal stability of LSP1 upon digoxin treatment (Figure 6G). Furthermore, Co‐IP analysis showed that digoxin treatment markedly disrupted the LSP1‐SOD1 interaction (Figure 6H). These findings collectively provide cellular evidence that digoxin directly engages LSP1 and disrupts its interaction with SOD1.

To determine whether the anti‐METs and antioxidant effects of digoxin are functionally dependent on LSP1, we compared the effects of digoxin in control versus si‐Lsp1 macrophages. As shown in Figure 6I–K, LPS stimulation induced METs formation, as evidenced by increased CitH3 expression and cfDNA levels, along with suppression of SOD1 activity. In control macrophages, digoxin treatment significantly reversed these changes. In contrast, si‐Lsp1 alone markedly reduced METs formation and restored SOD1 activity to levels comparable to those achieved by digoxin in control cells; importantly, the addition of digoxin to si‐LSP1 macrophages provided no further benefit beyond si‐Lsp1 alone. Collectively, these findings demonstrate that digoxin acts through LSP1 to suppress METs formation and restore redox homeostasis.

Furthermore, digoxin reduced intracellular ROS and mtROS levels (Figure 6L), indicating inhibition of the mtROS pathway. Intra‑articular injection of 100 nm digoxin into DMM‑induced OA mice alleviated cartilage damage (Figure 6M) and reduced osteophyte volume by micro‑CT analysis (Figure 6N), demonstrating protection against joint structural destruction. Digoxin also suppressed ROS accumulation in synovial tissue (Figure 6O,P), promoted COL2A1 expression while reducing MMP13 in cartilage (Figure 6Q), thereby mitigating cartilage damage and promoting matrix synthesis. Immunofluorescence showed that digoxin lowered synovial F4/80 and CitH3 signal (Figure 6R), confirming reduced METs formation in vivo.

To evaluate the potential toxicity of intra‐articular digoxin administration, we measured serum levels of ALT, AST, BUN, and Cr, and performed H&E staining of major organs (heart, lung, spleen, liver, and kidney) at the experimental endpoint (8 weeks post‐DMM). As shown in Figure 6F, there were no significant differences in any of the serum biochemical parameters among the Sham, DMM, DMM + Saline, and DMM + Digoxin groups. Consistently, histopathological examination revealed no signs of organ damage, inflammation, or structural abnormalities in any of the four groups (Figure 6G). These results demonstrate that intra‐articular injection of digoxin at the therapeutic concentration (100 nm) does not cause detectable systemic or organ‐specific toxicity in mice.

Collectively, digoxin blocks the LSP1‑SOD1 interaction, restores SOD1 activity, and inhibits NOX/mtROS‑mediated oxidative stress and METs release, which makes it a promising therapeutic candidate for OA through targeting the LSP1‑SOD1 axis (Figure 7A).

**FIGURE 7:**
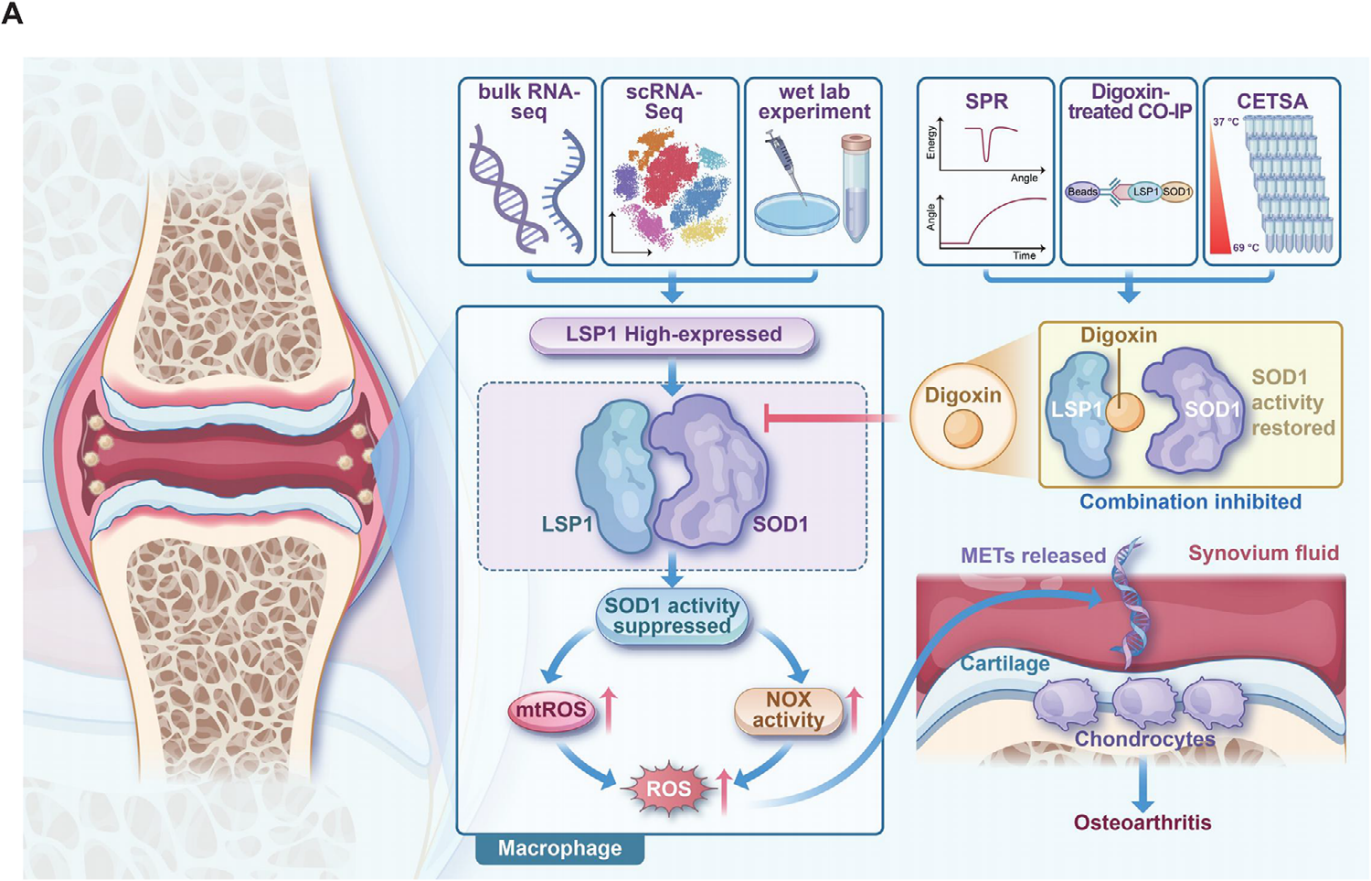
Schematic model of the LSP1–SOD1–METs pathogenic axis in OA and its therapeutic targeting by digoxin. (A) Upregulation of LSP1 in OA joint macrophages promotes disease by binding to and inhibiting SOD1, leading to ROS accumulation, activation of the NOX and mtROS axis, and METs‐driven cartilage destruction. The compound digoxin attenuates OA by competitively binding LSP1, restoring SOD1 activity, and suppressing METs formation in vitro and in vivo.

## 3 Discussion

This study elucidates a central mechanism by which synovial macrophages contribute to OA pathogenesis and identifies a repurposable therapeutic agent targeting this pathway. Synovial micro‐injury, leading to extracellular matrix exposure and innate immune activation, is a key initiating event in OA progression [43]. This initial damage triggers an inflammatory cascade that exacerbates synovitis [44]. Consistent with the central role of ECM dysregulation in OA [45, 46], we found OA‐upregulated genes predominantly enriched in ECM‐related pathways and specifically overexpressed in macrophages. A pathogenic feedback loop operates between synovial macrophages and chondrocytes: activated macrophages promote inflammation and ECM degradation, which in turn induce chondrocytes to produce inflammatory mediators that further amplify macrophage activation [47, 48]. Disrupting this loop therefore represents a promising therapeutic strategy for OA.

In the present study, we identified a significant upregulation of LSP1 specifically in macrophages in OA patients. This high‐LSP1 macrophage subpopulation was further enriched with gene sets related to METs. We also found that LSP1 upregulation did not induce classical M1/M2 polarization in macrophages. Together, these findings suggest that LSP1 upregulation in OA macrophages drives pathogenic METs formation. In addition to its classical cytosolic functions, emerging evidence suggests that LSP1 can also localize to the nucleus in certain cell types, such as endothelial cells, where it may associate with chromatin or transcription regulatory complexes. Whether a similar nuclear localization or transcription‐modulating function exists in macrophages remains unexplored in OA. While we cannot exclude such possibilities, our biochemical and functional data firmly establish that the cytosolic LSP1–SOD1 interaction is a key driver of the NOX/mtROS–METs axis in synovial macrophages.

SOD1 was prioritized from the IP‐MS hits for mechanistic follow‐up based on the following rationale. After excluding antibody contaminants and abundant structural proteins commonly observed as background in IP‐MS experiments—including actins, tubulins, keratins, myosin, and vimentin—SOD1 emerged as the only candidate with a direct enzymatic function in superoxide anion scavenging and a plausible mechanistic link to the redox imbalance underlying NOX and mtROS activation. It is well established that both NOX‐derived and mtROS pathways are essential for NETs formation, and our data indicated that LSP1‐driven METs similarly depend on these two oxidative pathways. Since SOD1 is a key enzyme that scavenges superoxide anions and maintains redox homeostasis, we reasoned that SOD1 inhibition by LSP1 would represent the most parsimonious mechanistic explanation linking LSP1 to the concurrent activation of both NOX and mtROS pathways. This hypothesis was subsequently confirmed by our biochemical and functional experiments. Our study reveals that LSP1 drives oxidative stress and METs release to accelerate OA. This interaction, mediated by a specific structural region of LSP1, suppresses SOD1 activity, thereby activating the NOX and mtROS pathways to promote METs formation. Collectively, these findings position LSP1‐mediated METs formation as a central pathogenic mechanism in OA.

Among the virtual screening hits from DrugBank, digoxin was selected for experimental validation because it displayed the highest binding affinity to LSP1 among the top candidates, and because its favorable safety profile as an FDA‐approved drug made it an attractive candidate for repurposing in OA. Other top‐ranking compounds with lower binding affinities or less favorable pharmacokinetic properties were not pursued further. Nevertheless, this screening result suggests that the LSP1‐SOD1 interface may be pharmacologically tractable through multiple distinct scaffolds (Table S10), providing a foundation for future drug repurposing efforts beyond digoxin. Our study demonstrated that digoxin binds to LSP1 with high affinity, competitively blocking its interaction with SOD1 and thereby restoring the antioxidant activity of SOD1. Consequently, this intervention abrogates the induction of METs from oxidatively stressed synovial macrophages. These findings suggest that digoxin, by targeting the LSP1‐SOD1 axis, represents a potential therapeutic candidate for osteoarthritis. Notably, a previous study reported that digoxin directly targets LRP4 in chondrocytes to exert chondroprotective effects [42]. Our findings do not contradict this; rather, they reveal a parallel and independent mechanism whereby digoxin targets LSP1 in synovial macrophages. Given that LRP4 is predominantly expressed in chondrocytes while LSP1 is haematopoietic‐restricted, digoxin appears to act as a dual‐target agent—simultaneously suppressing synovial inflammation (via LSP1) and preserving cartilage integrity (via LRP4). This convergence of distinct mechanisms highlights digoxin's unique potential for multi‐compartment disease modification in OA.

Several limitations of this study should be acknowledged. First, the upstream regulatory mechanisms governing LSP1 upregulation in OA macrophages remain undefined—including PU.1, NF‐κB/Sp1 crosstalk, and C/EBP family members [49, 50, 51]—which we have proposed as testable hypotheses for future investigation. Second, while our global knockout and bone marrow chimera experiments demonstrate that haematopoietic LSP1 drives OA pathology, a macrophage‐specific conditional knockout would provide definitive genetic proof of cell‐of‐origin specificity and help exclude potential contributions from other cell types. Third, although our study establishes the cytosolic LSP1–SOD1 interaction as a key pathogenic axis, LSP1 is known to undergo alternative splicing and may possess nuclear functions. Finally, digoxin may exert ancillary effects on transcription factors such as p53 and HIF [52, 53], and has been reported to interact with nuclear receptor superfamily members including RORγ [54]. While these additional mechanisms could represent alternative pathways contributing to digoxin's therapeutic effects in OA, they were not experimentally addressed in the present study and therefore warrant further investigation.

In conclusion, we elucidate a pathogenic cascade wherein LSP1(activator) ‑ SOD1 inhibition ‑ NOX/mtROS activation ‑ METs release ‑ cartilage degradation. On this basis, we successfully screened and validated that digoxin targets the LSP1‑SOD1 interaction, restores cellular redox homeostasis, effectively suppresses METs generation, and alleviates joint pathology. Collectively, this study identifies digoxin as a dual‐target therapeutic agent for OA: beyond its previously established chondroprotective effects, we demonstrate that digoxin alleviates joint pathology by suppressing synovial inflammation via targeting the LSP1–SOD1–METs axis. Together with prior reports, our findings position digoxin as a mechanistically distinct yet therapeutically convergent repurposable candidate, highlighting its unique multi‐target potential for disease modification in OA.

## 4 Materials & Methods

### 4.1 Data Acquisition and Bioinformatic Analysis

Gene Expression Omnibus (GEO) datasets were screened using keywords “osteoarthritis”, “Homo sapiens”, and “series”, yielding 47 initial datasets that were filtered by assay type, sample source, and annotation completeness (Table S1). Principal component analysis (PCA) verified data quality, and the microarray dataset GSE236924 (89 osteoarthritis and 7 controls) and single‐cell RNA‐sequencing (scRNA‐seq) dataset GSE216651 (3 osteoarthritis and 3 healthy controls) were selected for downstream analysis.

Differential expression analysis of GSE236924 was performed using the R package limma [55]. Gene Ontology (GO) and Kyoto Encyclopedia of Genes and Genomes (KEGG) enrichment analyses were conducted to annotate biological functions of differentially expressed genes (DEGs). Gene Set Enrichment Analysis (GSEA) [56] was implemented with the GSEABase package, using C5 GO gene sets from the Molecular Signatures Database (MSigDB) and calculating normalized enrichment scores (NES) to identify significantly enriched pathways in osteoarthritis.

Weighted gene co‐expression network analysis (WGCNA) [57] was applied to the top 25% most variable genes in GSE236924 to identify osteoarthritis‐associated modules. A suitable soft‐thresholding power was chosen to ensure scale‐free topology, and modules were detected using the dynamic tree‐cut algorithm. Hub genes were defined as genes with module membership (MM ≥ 0.8) and gene significance (GS ≥ 0.8) within key modules that overlapped with upregulated DEGs.

ScRNA‐seq data were processed using the Seurat package [58], including quality control, filtering, normalization, variable gene selection, and scaling. Batch effects were corrected using RunHarmony, followed by PCA and K‐nearest neighbor (KNN) clustering at a resolution of 0.3 determined by clustree. Cell clusters were annotated using canonical marker genes and visualized via UMAP. Macrophages were extracted for subclustering into eight subsets (C1–C8), with DEGs identified by FindAllMarkers and functionally annotated by GO enrichment. ClusterGVis was used to integrate DEG and pathway profiles for each subset.

High‐dimensional WGCNA (hdWGCNA) [59] was performed in macrophages to construct cell‐specific co‐expression modules using metacells derived from a KNN‐based similarity network. AUCell was applied to calculate gene set activity scores at the single‐cell level, with thresholds defined to label active cells, which were then projected onto UMAP plots.

Macrophage developmental trajectories were inferred by Monocle2 pseudotime analysis using DEGs across subsets as ordering genes [60]. Cell–cell communication patterns between control and osteoarthritis groups were analyzed using CellChat [61], with communication networks constructed and dominant signaling patterns extracted based on optimal pattern numbers determined by curve inflection. Interaction strength and pathway activity were visualized by comparative plots and heatmaps.

### 4.2 Animal Models and Experimental Protocols

Lsp1 knockout (Lsp1^−^/^−^) mice were generated via CRISPR/Cas9‐mediated gene targeting. Four sgRNAs targeting Lsp1 were microinjected into fertilized C57BL/6 embryos, and F0 heterozygotes were intercrossed to obtain homozygous mutants, with genotypes validated by PCR using strain‐specific primers.

To generate bone marrow chimeras, recipient C57BL/6 mice (CD45.1^+^ congenic) were lethally irradiated with 9 Gy. Bone marrow cells were harvested from femurs and tibias of donor mice (Lsp1^+^/^+^ or Lsp1^−^/^−^ on a CD45.2^+^ background). A total of 5 × 10^6^ donor bone marrow cells were injected intravenously into each recipient via the tail vein. Mice were allowed to recover for 8 weeks, and reconstitution efficiency was verified by flow cytometry analysis of peripheral blood CD45.1/CD45.2 chimerism before further experimentation.

Six‐ to eight‐week‐old C57BL/6 mice were housed under specific pathogen‐free conditions. Osteoarthritis was induced by destabilization of the medial meniscus (DMM) surgery on the left hind limb, with sham‐operated mice serving as controls. For intervention experiments, 24 mice were randomized into four groups: Sham, DMM + saline, DMM + DNase I, and DMM + PAD4 inhibitor (PADi). From postoperative day 2, mice received daily intra‐articular injections for 8 weeks (DNase I: 1 × 10^4^ IU/kg/day; PADi: 20 mg/kg/day). Rescue experiments were performed in Lsp1^−^/^−^ mice with daily intra‐articular LCS‐1 (20 mg/kg) or saline for 8 weeks starting on day 2 post‐DMM. A separate DMM group received intra‐articular digoxin (100 nm in 10 µL saline) daily for 8 weeks. All animal procedures were approved by the Animal Ethics Committee of Changhai Hospital (No.: CHEC(A.E)2024‐019).

### 4.3 Histological and Imaging Analyses

Human osteoarthritis (n = 12) and healthy control (n = 12) synovial tissues were obtained from Changhai Hospital with ethical approval and informed consent (No.: CHEC2025‐131, Table S7). Mouse synovial and cartilage tissues were fixed in formalin, paraffin‐embedded, and sectioned. Immunofluorescence staining was performed using primary and fluorophore‐conjugated secondary antibodies, with images captured by confocal microscopy. Co‐localization was analyzed using ImageJ. Pearson's correlation coefficient was calculated from at least three independent fields per sample. Immunohistochemistry (IHC) was conducted using HRP‐conjugated secondary antibodies and DAB chromogenic detection, followed by hematoxylyl counterstaining.

Hematoxylin and eosin (H&E) staining was used to evaluate general tissue morphology. Safranin O/Fast Green staining assessed cartilage proteoglycan content, and toluidine blue staining visualized chondrogenesis and matrix integrity. Mouse knee joints were scanned by micro‐CT (SkyScan 1276) at 10 µm resolution, with three‐dimensional reconstruction and quantitative analysis of bone microarchitecture. Mouse synovial tissues were prepared for transmission electron microscopy (TEM) using glutaraldehyde and osmium tetroxide fixation, ultrathin sectioning, and uranyl acetate–lead citrate staining.

Intracellular ROS levels were assessed using the fluorescent probe DCFH‐DA (2′,7′‐dichlorodihydrofluorescein diacetate). Cells were incubated with 10 µm DCFH‐DA for 30 min at 37°C in the dark, washed, and then analyzed by fluorescence microscopy or flow cytometry. ROS levels in synovial tissues were detected by dihydroethidium (DHE) staining. Frozen sections were incubated with 10 µm DHE for 30 min at 37°C, washed, and imaged by confocal microscopy. Mitochondrial superoxide levels were assessed using the MitoSOX Red fluorescent probe (a fluorogenic indicator for mitochondrial superoxide). Cells were incubated with 5 µm MitoSOX Red for 15 min at 37°C in the dark, washed, and analyzed by flow cytometry or fluorescence microscopy.

### 4.4 Cell Culture and Molecular Biology

RAW264.7 and HEK293T cells were cultured in DMEM with 10% fetal bovine serum (FBS) and antibiotics. A Transwell coculture system was established using macrophages (upper chamber) and chondrocytes (lower chamber), with macrophages stimulated by LPS (100 ng/mL) and treated with DNase I or PADi.

For preparation of METs‐containing medium (used in Figure 4A), RAW264.7 macrophages were stimulated with LPS (100 ng/mL) for 12 h. The supernatant was removed, wells were gently washed with PBS, and adherent METs were detached by incubation with micrococcal nuclease (MNase, 20 U/mL) for 30 min at 37°C. The resulting suspension was centrifuged (350 × g, 5 min), and the supernatant was collected as METs^+^ medium. METs^−^ medium was prepared from unstimulated macrophages processed in parallel. dsDNA content was quantified using the QuantiFluor dsDNA system to normalize METs concentration. This protocol was adapted from the method described for NETs collection by Chen et al. [62].

RAW264.7 cells were transfected with Lsp1 siRNA at gradient concentrations or Lsp1‐overexpressing plasmids, while HEK293T cells were transfected with siRNA or mutant LSP1 constructs (R221A, S232A, and 206–242 truncation) via Lipofectamine 3000 or jetPRIME. Stable Lsp1‐overexpressing cells were selected with neomycin. Primary mouse chondrocytes were isolated from knee cartilage via type II collagenase digestion and filtered to obtain single‐cell suspensions.

Flow cytometry was used to quantify LSP1^+^ macrophage proportions in mouse synovial tissues. Superoxide dismutase (SOD) and NADPH oxidase (NOX) activities were measured using commercial ELISA kits. Western blotting was performed following protein extraction and BCA quantification, with band intensities quantified by ImageJ. Immunoprecipitation coupled with mass spectrometry (IP‐MS) was processed by in‐gel digestion and LC‐MS/MS, with protein identification at FDR < 1%. Co‐immunoprecipitation (Co‐IP) was conducted using Protein A/G magnetic beads, followed by standard Western blotting. CETSA was performed to validate the direct binding between digoxin and LSP1 in living cells. Cells were treated with digoxin (100 nm) or vehicle for 6 h, lysed, incubated with digoxin or vehicle, and heated at 37–69°C (4°C increments) for 10 min. Soluble fractions were analysed by Western blotting, and thermal stability curves were plotted as the percentage of remaining LSP1 relative to the lowest temperature.

Total RNA was extracted using TRIzol for reverse transcription quantitative PCR (RT‐qPCR), with relative expression calculated by the 2^−^ΔΔCt method using GAPDH as a reference. Primer sequences are listed in Table S8. Surface plasmon resonance (SPR) was performed at 25°C using a CM5 sensor chip. The target protein was immobilized via amine coupling, and remaining active groups were blocked with ethanolamine‐HCl. Small‐molecule inhibitors were dissolved in running buffer (10 mm HEPES, pH 7.4, 150 mm NaCl, 0.005% Tween 20) and filtered. Serial dilutions of inhibitors were injected at 30 µL/min. Association and dissociation were monitored in real time. Sensorgrams were fitted with a 1:1 binding model using BIAcore Evaluation Software to obtain k_a_, k_d, and K_D. LSP1 three‐dimensional structure was predicted by SWISS‐MODEL homology modeling, and molecular docking was performed using AutoDock, with optimal conformations visualized by PyMOL and Discovery Studio.

### 4.5 Statistical Analysis

Statistical analyses were performed using R 4.4, GraphPad Prism 9.5, ImageJ, and FlowJo. Data are presented as mean ± standard deviation (SD) from at least three independent replicates. Student's t‐test or Wilcoxon rank‐sum test was used for two‐group comparisons, and one‐ or two‐way ANOVA for multiple groups. A *p*‐value < 0.05 was considered statistically significant.

## Author Contributions

Design and performance of the experiments, collection and analysis of the data, and draft of the manuscript, YP and XF. Modification of study design and manuscript revision, CL. Drawing and typesetting, JT. Collection of samples, ZL and CL. Performance of statistical analysis, YD and CM. Design of the study, YX. Manuscript revision and funding acquisition, WX. All authors read and approved the final manuscript.

## Funding

This research was supported by the National Natural Science Foundation of China (82402766, 82172390); The First Affiliated Hospital of Naval Medical University Clinical Medical Research (2024LYB06).

## Ethics Statement

The acquisition and use of human tissue samples were approved by the Changhai Hospital Medical Ethics Committee (No.: CHEC2025‐131), and written informed consent was obtained from all participants prior to their inclusion in the study. All animal procedures were approved by the Animal Ethics Committee of Changhai Hospital (No.: CHEC(A.E)2024‐019).

## Conflicts of Interest

The authors declare no conflicts of interest.

## Supporting information




**Supporting File**: advs78097‐sup‐0001‐SuppMat.docx.

## Data Availability

All data relevant to the study are included in the article. Not applicable.
