# Supplementary material for: Digoxin Alleviates Osteoarthritis by Inhibiting Macrophage Extracellular Trap Formation via Disruption of the LSP1‐SOD1 Interaction
Source: Adv Sci (Weinh). 2026 Sep 30:e78097. Online ahead of print. doi: 10.1002/advs.78097 (PMC13626028; doi:10.1002/advs.78097)
Supplement: Supplementary file 1 [file ADVS-9999-e78097-s001.docx]

**Supplementary Figure 1. Initial bioinformatics analysis of the OA synovial transcriptome dataset and single-cell RNA sequencing dataset.**

(A) Principal component analysis (PCA) of transcriptomic data from dataset GSE236924.

(B) Gene set enrichment analysis (GSEA) of upregulated DEGs showed significant enrichment for extracellular matrix structure formation pathways.

(C) GSEA reveals enrichment of OA-upregulated genes in extracellular matrix and macrophage activation pathways compared to healthy controls

(D) Determination of the soft‑thresholding power (β = 5) in WGCNA based on scale independence and mean connectivity criteria.

(E) Cluster dendrogram of modules identified using the Dynamic Tree Cut algorithm.

(F) Pseudobulk analysis of single‑cell transcriptomic data was performed to compare the expression trends of five key genes between the OA and control groups.

(G) UMAP visualization of cells after Harmony integration, demonstrating effective removal of inter-sample batch effects.

(H) UMAP plot showed unsupervised clustering of all synovial cells prior to annotation.

(I) Marker genes for each cell subpopulation were identified using the COSG algorithm and visualized in a dot plot. In the plot, dot size represents the proportion of cells expressing the marker gene within its respective subpopulation, while color intensity indicates the average expression level of the gene.

(J) Feature plots of canonical marker genes used for cell type annotation.

(K) Bar plot depicted the proportional composition of major cell types across the six individual samples.

(L) Volcano plots showed differentially expressed genes for each annotated cell cluster.

**Supplementary Figure 2. In-depth bioinformatics characterization of synovial macrophage subpopulations and their communication.**

(A) Soft‑thresholding power (β = 3) selected for hdWGCNA of macrophages. -
(B) The hub genes of each module from the hdWGCNA analysis, as well as the UMAP co‑expression network plot for the LSP1 gene, were presented.
(C) The expression patterns of different hMEs (module eigengenes) across various cell populations were presented.
(D) Determination of the soft‑thresholding power (β = 3) in hdCNA based on scale independence and mean connectivity criteria.

(E) Threshold determination for AUCell analysis using the METs gene set. An AUC threshold of 0.57 identified 1210 cells with significant METs pathway activity.
(F) Number of genes detected by cells of AUCell analysis.

(G) AUCell analysis for M1 polarization gene sets, revealing minimal enrichment above the threshold in macrophage subsets.

(H) AUCell analysis of M2 polarization gene sets showed little enrichment above the threshold in macrophage subsets.

(I) BEAM analysis of macrophage pseudotime reveals bifurcating differentiation trajectories with divergent *LSP1* expression: low in Branch 1 and high in Branch 2.

(J) 	Barplot depicted enhanced interaction strength of key inflammatory pathways (e.g., IL-1, TNF, CXCL) emanating from macrophages in OA compared to controls.

(K) Differentiation trajectories bifurcate into two branches, with C1 on one branch and C2 giving rise to terminal states C5/C6 on the other.

(L) Cell communication analysis shows stronger overall signaling activity in subpopulations C5 and C6 from the OA group compared to controls.

(M-N) Circleplots showed increased ingoing and outgoing interaction strength from C5 and C6 macrophage subsets in OA.

**Supplementary Figure 3. Optimization and validation of LSP1 knockdown and overexpression in RAW264.7 macrophages.**

(A) Screening of siRNA concentrations for optimal Lsp1 knockdown. 50 nM was selected for subsequent experiments.

(B) Western blot confirmed LSP1 knockdown at the protein level using 50 nM siRNA.

(C-D) Immunofluorescence was performed to analyze the expression of CitH3 in RAW264.7 cells following LPS treatment over 0, 2, 4, 6, 12, and 24 h.

**Supplementary Figure 4. Experimental optimization and in vivo validation of LSP1 modulation.**

(A) Validation of Lsp1 overexpression at the RNA level by RT-qPCR.

(B) Western blot analysis of LSP1 overexpression at the protein level.

(C) The Western blot was used to explore the expression of Control cell and Lsp1 overexpressing RAW264.7 cells following LPS treatment over 0, 6, 12, and 24 h.

(D) The levels of cfDNA from Control cell and Lsp1 overexpressing RAW264.7 cells with or without LPS treatment.

(E) Schematic of the workflow for analyzing the proportion of synovial LSP1⁺ macrophages in model and sham‑operated mice.

(F) Genotyping of Lsp1^-/-^ mice was performed by PCR. Wild‑type mice showed only a 368 bp band (primers p3/p4); heterozygous mice (Lsp1^+/-^) displayed both a 501 bp band (primers p1/p2) and a 368 bp band (primers p3/p4); homozygous knockout mice (Lsp1^-/-^) showed only the 501 bp band (primers p1/p2).

**Supplementary Figure 5. LSP1-mediated METs formation in human THP-1 cells depends on NOX and mtROS pathways**

(A) NOX activity assay in RAW264.7 macrophages transfected with Lsp1-overexpressing plasmids (Lsp1-OE) or vector controls, followed by LPS stimulation (100 ng/mL) with or without VAS2870 (NOX inhibitor) treatment.

(B) NOX activity assay in raw264.7 macrophages transfected with Lsp1-overexpressing plasmids (Lsp1-OE) under different treatment conditions: untreated control (Lsp1-OE alone), Lsp1-OE + LPS + VAS2870 + Mito-TEMPO (dual inhibition), Lsp1-OE + LPS + VAS2870 (NOX inhibition alone), and Lsp1-OE + LPS + Mito-TEMPO (mtROS inhibition alone).

(C) NOX activity assay in THP-1 macrophages transfected with LSP1-overexpressing plasmids (LSP1-OE) or vector controls, followed by LPS stimulation (100 ng/mL) with or without VAS2870 (NOX inhibitor) treatment.

(D) NOX activity assay in THP-1 macrophages transfected with LSP1-overexpressing plasmids (LSP1-OE) under different treatment conditions: untreated control (LSP1-OE alone), LSP1-OE + LPS + VAS2870 + Mito-TEMPO (dual inhibition), LSP1-OE + LPS + VAS2870 (NOX inhibition alone), and LSP1-OE + LPS + Mito-TEMPO (mtROS inhibition alone).

(E,F) WB analysis of CitH3 in LSP1-OE THP-1 cells with LPS stimulation and NOX inhibitor VAS2870 treatment (E); quantitative analysis of CitH3/GAPDH (F).

(G,H) WB analysis of CitH3 in LSP1-OE THP-1 cells with LPS stimulation and mtROS inhibitor Mito-TEMPO treatment (G); quantitative analysis of CitH3/GAPDH (H).

(I,J) WB analysis of CitH3 in LSP1-OE THP-1 cells with LPS stimulation and combined VAS2870+Mito-TEMPO treatment (I); quantitative analysis of CitH3/GAPDH (J).

**Supplementary Figure 6. Determination of the optimal digoxin concentration for inhibiting METs formation *in vitro*.**

(A-B) Immunofluorescence analysis of CitH3 expression in LPS‑stimulated RAW264.7 cells treated with increasing concentrations of digoxin (0, 12.5, 25, 50, 100, and 200 nM) for 6 h.

(C) Western blot analysis of CitH3 expression in LPS‑stimulated RAW264.7 cells treated with increasing concentrations of digoxin (0, 12.5, 25, 50, 100, and 200 nM) for 6 h.

(D) WB analysis of CitH3 in THP-1 cells with LPS stimulation and combined digoxin treatment.

(E) Western blot analysis of p‑H3 expression in RAW264.7 macrophages with LPS stimulation and digoxin treatment.

(F) Serum ALT, AST, BUN, and Cr levels in Sham, DMM, DMM + Saline, and DMM + Digoxin groups (n = 6 per group).

(G) Representative H&E staining of heart, lung, spleen, liver, and kidney sections from the same groups.

**SUPPLEMENTARY TABLES**

**Table S1. Overview of knee osteoarthritis-related datasets retrieved from the GEO database.**

| Series | Experiment type | Tissue |
| --- | --- | --- |
| GSE104113 | Array | chondrocytes |
| GSE109449 | High throughput sequencing | synovial fibroblasts |
| GSE143514 | High throughput sequencing | synovial tissues |
| GSE158875 | High throughput sequencing | chondrocytes |
| GSE163552 | High throughput sequencing | pbmc |
| GSE168505 | High throughput sequencing | cartilage |
| GSE169077 | Array | cartilage |
| GSE174049 | Array | cartilage |
| GSE175962 | Array | cartilage |
| GSE176199 | High throughput sequencing | chondrocytes、normal cartilage stromal cells、cartilage mesenchymal stromal cells |
| GSE176223 | High throughput sequencing | synovial tissues |
| GSE178092 | Array、high throughput sequencing | cartilage |
| GSE178557 | Array | cartilage |
| GSE178724 | Array | cartilage |
| GSE185064 | High throughput sequencing | meniscus |
| GSE19060 | Array | cells isolated from meniscus |
| GSE205196 | High throughput sequencing | synovial tissues |
| GSE206848 | Array | synovial tissues |
| GSE213070 | High throughput sequencing | synovial tissues |
| GSE214394 | High throughput sequencing | cartilage(Hip & Knee) |
| GSE217012 | High throughput sequencing | synovial fibroblasts |
| GSE221415 | High throughput sequencing | cartilage |
| GSE235610 | High throughput sequencing | cartilage |
| GSE236924 | Array | joint tissue |
| GSE243821 | Array | Fibroblast-like synoviocytes |
| GSE246425 | High throughput sequencing | chondrocytes(young & old) |
| GSE252542 | Array | macrophages |
| GSE254682 | High throughput sequencing | synovial tissues |
| GSE264185 | High throughput sequencing | cartilage chondrocytes |
| GSE32317 | Array | synovial tissues |
| GSE36700 | Array | synovial tissues |
| GSE45233 | Array | meniscus |
| GSE46750 | Array | synovial tissues |
| GSE51588 | Array | subchohndral bone(lateral & medial) |
| GSE54749 | Array | cartilage |
| GSE61385 | Array | ACL |
| GSE63106 | Array | cartilage |
| GSE63695 | Array | chondrocyte |
| GSE64394 | Array | chondrocyte |
| GSE66635 | Array | normal cartilage |
| GSE73764 | Array | Fibroblast-like synoviocytes |
| GSE7669 | Array | synovial fibroblasts |
| GSE82107 | Array | synovial tissues |
| GSE98460 | Array | cartilage(medial &lateral) |
| GSE98918 | Array | meniscus |
| GSE99662 | High throughput sequencing | synovial tissues |
| GSE283079 | High throughput sequencing | synovial tissues |

**Table S2. Top 50 differentially expressed genes from microarray analysis.**

| symbol | logFC | AveExpr | t | P.Value | adj.P.Val |
| --- | --- | --- | --- | --- | --- |
| FOS | 2.16757 | 14.19601 | 16.01102762 | 5.75494872793949e-29 | 3.49613135222324e-25 |
| CHID1 | 1.154857 | 10.4534 | 14.05445245 | 4.06408021004487e-25 | 5.84746277590009e-22 |
| COL1A2 | 1.215584 | 14.75068 | 14.02413543 | 4.6792951420116e-25 | 6.39601154723711e-22 |
| COL3A1 | 1.650964 | 14.62563 | 13.93638722 | 7.04089786915968e-25 | 9.06243195368076e-22 |
| TRAPPC1 | 1.198343 | 10.53778 | 13.93356338 | 7.13419403400447e-25 | 9.06243195368076e-22 |
| LSP1 | 1.339342 | 10.83757 | 13.48619113 | 5.80790623889399e-24 | 5.38215717985643e-21 |
| ALDH18A1 | 1.188675 | 10.53388 | 13.18114882 | 2.45744431315308e-23 | 2.03576920941885e-20 |
| CDK14 | 1.324392 | 11.10628 | 13.084779 | 3.8841670713307e-23 | 3.12304168566185e-20 |
| B3GALNT1 | 2.005935 | 8.485006 | 13.05152583 | 4.54986710011677e-23 | 3.60527512607079e-20 |
| PCOLCE | 1.602722 | 13.05672 | 13.03788428 | 4.85508165479941e-23 | 3.73875478135433e-20 |
| TMEM119 | 2.462009 | 10.59229 | 12.81933871 | 1.37745665652345e-22 | 9.29783243153326e-20 |
| PLD3 | 1.243538 | 11.50772 | 12.6075809 | 3.80088946057529e-22 | 2.16678542510822e-19 |
| CALR | 1.710304 | 9.239886 | 12.51048701 | 6.06230029609064e-22 | 3.18707950662265e-19 |
| MAGED1 | 1.230353 | 12.23905 | 12.44001202 | 8.51232944326374e-22 | 4.34964123654621e-19 |
| OLFML2B | 2.111368 | 11.88893 | 12.33091691 | 1.44091508970852e-21 | 6.89696761231092e-19 |
| KDELR2 | 1.008626 | 12.54229 | 12.23328034 | 2.31004897548501e-21 | 1.05251606445536e-18 |
| MRC2 | 1.143033 | 9.921224 | 12.17181006 | 3.11075460178412e-21 | 1.31845354924455e-18 |
| NUCB1 | 1.472499 | 10.3085 | 12.06215146 | 5.29404319896674e-21 | 2.11278694820078e-18 |
| SLC30A5 | 1.325727 | 8.740509 | 12.03673226 | 5.98935965980859e-21 | 2.3623509489635e-18 |
| TFCP2 | 1.122726 | 8.324202 | 11.99510206 | 7.33168271282932e-21 | 2.75175425292428e-18 |
| MAZ | 1.059104 | 8.568794 | 11.91615761 | 1.0762647884035e-20 | 3.7964372455459e-18 |
| CCDC80 | 1.842809 | 12.30504 | 11.86492083 | 1.38115124877509e-20 | 4.63278800777779e-18 |
| HACD3 | 1.148519 | 9.326878 | 11.81356613 | 1.77380423796477e-20 | 5.73862406572331e-18 |
| IDUA | 1.217209 | 8.215261 | 11.73091546 | 2.6545021212239e-20 | 7.93086904250912e-18 |
| TBC1D24 | 1.149349 | 9.099781 | 11.63480885 | 4.24477262952549e-20 | 1.21509394512726e-17 |
| NAP1L3 | 1.947572 | 9.746823 | 11.60272678 | 4.96567366186781e-20 | 1.39947529619908e-17 |
| ATP6V1A | 1.967343 | 9.539303 | 11.55589152 | 6.24437464998898e-20 | 1.68182849255245e-17 |
| TFDP1 | 1.08125 | 8.869643 | 11.53032117 | 7.07697724805081e-20 | 1.86025832229413e-17 |
| TMCO3 | 1.741609 | 9.906239 | 11.39113066 | 1.39991985207937e-19 | 3.34238506167858e-17 |
| THY1 | 2.431705 | 12.09483 | 11.2907982 | 2.29093639984792e-19 | 5.13348146154446e-17 |
| INPP4A | 1.13117 | 8.52161 | 11.23258905 | 3.04963749189814e-19 | 6.66955719478123e-17 |
| NAB1 | 2.203325 | 7.727264 | 11.2275294 | 3.12644976066767e-19 | 6.7832793914486e-17 |
| GPC6 | 2.261663 | 9.933448 | 11.15076233 | 4.56088142751272e-19 | 9.44568909277492e-17 |
| SCRG1 | 3.244966 | 11.62899 | 11.1120788 | 5.51757607601008e-19 | 1.11318624337952e-16 |
| PRMT6 | 1.393679 | 8.843982 | 11.06480558 | 6.96409058379236e-19 | 1.34071004460862e-16 |
| EPB41L2 | 1.426982 | 10.75816 | 11.01815361 | 8.76410456268681e-19 | 1.62152006036724e-16 |
| DNAJC10 | 1.205815 | 9.273565 | 11.00855481 | 9.18876930037128e-19 | 1.68025405183211e-16 |
| CREB1 | 1.134734 | 8.901838 | 10.82369066 | 2.2880034505649e-18 | 3.79080571695867e-16 |
| GMDS | 1.305865 | 8.590333 | 10.81703224 | 2.36451189810204e-18 | 3.90573075615495e-16 |
| GOLGA2 | 1.020044 | 9.492182 | 10.79792001 | 2.59866250150642e-18 | 4.22862715088879e-16 |
| CDH11 | 2.247552 | 10.86232 | 10.71148777 | 3.98383055641467e-18 | 6.2055822128767e-16 |
| SLC39A6 | 1.366699 | 8.945963 | 10.70895544 | 4.03403394842679e-18 | 6.24818147677719e-16 |
| CD47 | 1.097207 | 12.03435 | 10.70549497 | 4.10366409353977e-18 | 6.33807441565783e-16 |
| YIPF5 | 1.010374 | 10.02867 | 10.68461335 | 4.55014857073758e-18 | 6.94914450014181e-16 |
| P4HB | 2.049876 | 11.68566 | 10.5972911 | 7.00947767130685e-18 | 9.95436861503123e-16 |
| LMNA | 1.265629 | 12.20838 | 10.53166348 | 9.70097863761823e-18 | 1.31613153104659e-15 |
| EMILIN1 | 1.354467 | 9.836272 | 10.51657078 | 1.04540013380266e-17 | 1.41002560970847e-15 |
| KDELR3 | 1.533113 | 11.89651 | 10.5162539 | 1.04704233661022e-17 | 1.41002560970847e-15 |
| FKBP7 | 1.782892 | 7.295593 | 10.49337397 | 1.17270942486931e-17 | 1.55249123013874e-15 |
| ENTPD1 | 1.397673 | 9.501005 | 10.46957879 | 1.31945610986197e-17 | 1.70950859731524e-15 |

**Table S3. Marker genes identified by COSG for all clusters.**

|  | Endothelial cell | Fibroblast | Smooth muscle cell | T cell | Macrophage | Mast cell | Monocyte | B cell | Plasma cell | Dendritic cell |
| --- | --- | --- | --- | --- | --- | --- | --- | --- | --- | --- |
| 1 | VWF | DCN | ACTA2 | CCL5 | F13A1 | TPSAB1 | TIMD4 | MS4A1 | MZB1 | CLEC9A |
| 2 | CCL14 | C1S | NOTCH3 | GZMA | MRC1 | TPSB2 | FCGR3A | TNFRSF13C | DERL3 | XCR1 |
| 3 | PLVAP | C1R | MYH11 | NKG7 | LYVE1 | CPA3 | FBP1 | VPREB3 | JSRP1 | PPY |
| 4 | ACKR1 | PLA2G2A | PLN | CD3E | CXCL8 | MS4A2 | TREM2 | IGHD | JCHAIN | CLNK |
| 5 | ADGRL4 | TNXB | MCAM | CST7 | MS4A6A | RHEX | MARCO | CD79A | TNFRSF17 | CCDC26 |
| 6 | PECAM1 | LTBP4 | MUSTN1 | CD3D | STAB1 | SLC18A2 | NCF1 | SHISA8 | AC012236.1 | SEPTIN3 |
| 7 | EMCN | SCARA5 | TPM2 | TRBC2 | CD83 | RGS13 | ABCC3 | BLK | FCRL5 | LGALS2 |
| 8 | CLEC14A | EFEMP1 | CARMN | GZMM | CSF1R | CTSG | ITLN1 | SPIB | IGHG1 | IDO1 |
| 9 | ECSCR | LUM | PPP1R14A | CD2 | CLEC10A | KIT | S100A8 | PAX5 | SPAG4 | IDO2 |
| 10 | RAMP3 | COL1A2 | RERGL | TRAC | PLEK | CALB2 | LINC01094 | LINC01781 | FAM92B | WDFY4 |
| 11 | EGFL7 | CCN5 | MYLK | IL32 | IL1B | HDC | FFAR4 | LINC01857 | AMPD1 | LINC01357 |
| 12 | TM4SF1 | COL6A2 | RRAD | BCL11B | FCGR2A | GCSAML | FPR3 | BCL11A | IGHG4 | BX664727.3 |
| 13 | PCAT19 | NOVA1 | RCAN2 | ACAP1 | FCGR2B | RAB27B | SMIM25 | TNFRSF13B | IGHG3 | TREML2 |
| 14 | SOX18 | ABI3BP | TBX2 | GZMK | MPEG1 | HECW2-AS1 | LILRB4 | CD19 | POU2AF1 | BTLA |
| 15 | ADCY4 | PCOLCE2 | LMOD1 | GZMH | CXCL3 | SIGLEC6 | IFI30 | FCRLA | IGHE | LINC02206 |
| 16 | MMRN2 | LRP1 | HES4 | TRBC1 | MS4A7 | AL157895.1 | CXADR | CD22 | IGLC3 | FLT3 |
| 17 | CLDN5 | MMP2 | CNN1 | LCK | FCER1G | HPGDS | LY86 | FCRL2 | SDC1 | SNX22 |
| 18 | NPDC1 | SERPING1 | SYNPO2 | IL7R | MS4A4A | HPGD | ACP5 | BANK1 | IGLC1 | PADI2 |
| 19 | PTPRB | CFD | TAGLN | GZMB | CCL3 | IL1RL1 | CYBB | NIBAN3 | IGKV1-39 | UPK3A |
| 20 | C2CD4B | MEG3 | RGS5 | CD3G | CD163 | ADCYAP1 | SLC16A3 | CXCR5 | IGLV6-57 | SEMA7A |
| 21 | CD93 | FBLN1 | ACTG2 | PRF1 | CD14 | AL078590.2 | HOXB6 | LINC02397 | RASSF6 | HLA-DOB |
| 22 | FLT1 | IGFBP6 | FHL5 | CD247 | GPR183 | SLC45A3 | MSR1 | FAM30A | PNOC | RUBCNL |
| 23 | HYAL2 | ISLR | GJA4 | CD52 | TYROBP | KRT19 | LST1 | TLR10 | IGHG2 | RAB7B |
| 24 | CDH5 | FSTL1 | KCNAB1 | RUNX3 | AIF1 | FCER1A | SLC7A7 | LY9 | IGLC2 | SLAMF7 |
| 25 | NRN1 | MGP | COX4I2 | CD48 | EMB | LIF | CAPG | IGHM | DPEP1 | PPM1J |
| 26 | CYYR1 | PLAC9 | CASQ2 | CXCR4 | BCL2A1 | VWA5A | IGSF21 | P2RX5 | CD79A | FUT7 |
| 27 | DIPK2B | CYBRD1 | ADRA2A | CTSW | NLRP3 | AL355881.1 | L1TD1 | CD24 | IGKC | TACSTD2 |
| 28 | SLC9A3R2 | PRG4 | AVPR1A | CD7 | CFP | GATA2 | LILRB5 | STAP1 | DNAAF1 | SLAMF8 |
| 29 | ITGB4 | MGST1 | ITGA8 | KLRB1 | FOLR2 | GPR65 | AC026369.3 | AIM2 | IGHA2 | ASB2 |
| 30 | SELE | COL6A3 | EDNRA | IL2RG | C1QA | CD69 | AP1B1 | FCMR | IGHA1 | DUSP4 |
| 31 | FAM110D | GPX3 | NEXN | LIME1 | CCL3L1 | MAOB | LYZ | SELL | FAM30A | CADM1 |
| 32 | ADAMTS9 | S100A13 | MRVI1 | CORO1A | RNASE6 | GAPT | VSIG4 | GPR18 | LINC02362 | MYCL |
| 33 | RASIP1 | PDGFRA | TBX2-AS1 | KLRK1 | C5AR1 | LAX1 | GAL3ST4 | LINC00926 | CD27 | PAK3 |
| 34 | MYCT1 | MXRA8 | GUCY1A1 | GNLY | IQGAP2 | AREG | MYO1G | CCR7 | SMPDL3B | PKP2 |
| 35 | RBP7 | CCDC80 | ITGA7 | TBC1D10C | CD209 | IL18R1 | ITGAM | FCER2 | QPCT | GCSAM |
| 36 | PODXL | CFH | EFHD1 | HCST | CTSS | TNIK | C1QB | HLA-DOB | CD38 | CYP2S1 |
| 37 | LIFR | PCOLCE | WTIP | CD8A | SCN9A | BATF | DLEU7 | ADAM28 | ZBP1 | VMO1 |
| 38 | JAM2 | COL6A1 | CLMN | KLRD1 | TNF | PTGS1 | HAVCR2 | RALGPS2 | U62317.4 | AL118508.1 |
| 39 | SOX7 | CRLF1 | TPM1 | PTPN7 | C1QC | LTC4S | C2 | POU2AF1 | BRSK1 | GPR157 |
| 40 | SHANK3 | FBN1 | TINAGL1 | RAC2 | C1QB | GMPR | HK3 | LTB | WNT10A | S100B |
| 41 | AQP1 | LTBP3 | MAP1B | CD69 | GPR34 | SMYD3 | CD84 | GAPT | IGHM | C1orf54 |
| 42 | ERG | FN1 | ISYNA1 | HOPX | NCF4 | ACOT7 | ENHO | CD79B | PIM2 | IRF8 |
| 43 | PLAT | NNMT | MYL9 | DUSP2 | SIGLEC1 | TMEM233 | GSDMA | PLD4 | MEI1 | BATF3 |
| 44 | KANK3 | ANGPTL2 | FRZB | XCL2 | P2RY14 | PTGS2 | LINC02611 | BCAS4 | AC078883.1 | BCL11A |
| 45 | SPNS2 | MRC2 | CRISPLD2 | SPOCK2 | RGL1 | SAMSN1 | LTA4H | COCH | IL5RA | TSPAN33 |
| 46 | PALM2-AKAP2 | PRELP | PDE5A | SKAP1 | LYZ | BIRC3 | C1QC | CPNE5 | TRPV3 | AP1S3 |
| 47 | TIE1 | SDC2 | ARHGEF17 | CD96 | LCP2 | RAC2 | KCNQ3 | IGHA2 | TMC3-AS1 | DNASE1L3 |
| 48 | S1PR1 | GSN | GUCY1B1 | ARL4C | SLC40A1 | STX3 | LAIR1 | SP140 | HLA-DOB | DAPP1 |
| 49 | TSPAN7 | TIMP2 | CDH6 | PYHIN1 | HCLS1 | TMEM154 | SLC11A1 | IGHA1 | SLAMF7 | PPP1R3G |
| 50 | GIMAP7 | RNASE4 | LDB3 | PRKCQ-AS1 | RBM47 | STMN1 | LAPTM5 | CD37 | LINC01480 | C15orf48 |

**Table S4.  Differentially expressed genes for all clusters.**

| gene | p_val | avg_log2FC | pct.1 | pct.2 | p_val_adj | cluster |
| --- | --- | --- | --- | --- | --- | --- |
| CD79A | 0 | 3.869337 | 0.865 | 0.005 | 0 | B cell |
| MS4A1 | 0 | 3.694547 | 0.782 | 0.002 | 0 | B cell |
| CD52.1 | 0 | 3.195529 | 0.859 | 0.077 | 0 | B cell |
| LTB.1 | 0 | 2.924732 | 0.75 | 0.025 | 0 | B cell |
| IGHM | 0 | 2.81233 | 0.442 | 0.015 | 0 | B cell |
| LGALS2 | 0 | 3.370418 | 0.926 | 0.01 | 0 | Dendritic cell |
| WDFY4 | 0 | 2.549863 | 0.944 | 0.016 | 0 | Dendritic cell |
| CLEC9A | 0 | 2.228127 | 0.833 | 0.001 | 0 | Dendritic cell |
| IDO1 | 0 | 1.952221 | 0.815 | 0.01 | 0 | Dendritic cell |
| XCR1 | 0 | 1.483792 | 0.5 | 0.001 | 0 | Dendritic cell |
| ACKR1 | 0 | 4.292557 | 0.7 | 0.06 | 0 | Endothelial cell |
| CCL14 | 0 | 3.940357 | 0.742 | 0.05 | 0 | Endothelial cell |
| VWF | 0 | 3.741161 | 0.839 | 0.033 | 0 | Endothelial cell |
| TM4SF1 | 0 | 3.493264 | 0.798 | 0.134 | 0 | Endothelial cell |
| SELE | 0 | 3.257865 | 0.423 | 0.02 | 0 | Endothelial cell |
| PRG4.1 | 0 | 3.716835 | 0.899 | 0.647 | 0 | Fibroblast |
| PLA2G2A.1 | 0 | 3.687002 | 0.913 | 0.433 | 0 | Fibroblast |
| DCN.1 | 0 | 3.52353 | 0.963 | 0.463 | 0 | Fibroblast |
| APOD.1 | 0 | 3.366508 | 0.567 | 0.356 | 0 | Fibroblast |
| CRTAC1.1 | 0 | 3.099517 | 0.534 | 0.233 | 0 | Fibroblast |
| CXCL8.1 | 0 | 3.815664 | 0.596 | 0.116 | 0 | Macrophage |
| CCL3 | 0 | 3.386333 | 0.53 | 0.095 | 0 | Macrophage |
| IL1B | 0 | 3.355953 | 0.364 | 0.036 | 0 | Macrophage |
| S100A9.1 | 0 | 3.335268 | 0.587 | 0.088 | 0 | Macrophage |
| RNASE1.3 | 0 | 3.327918 | 0.812 | 0.26 | 0 | Macrophage |
| TPSB2 | 0 | 6.51547 | 0.953 | 0.013 | 0 | Mast cell |
| TPSAB1 | 0 | 6.080638 | 0.939 | 0.008 | 0 | Mast cell |
| CPA3 | 0 | 4.539008 | 0.826 | 0.003 | 0 | Mast cell |
| HPGDS | 0 | 3.843881 | 0.795 | 0.044 | 0 | Mast cell |
| MS4A2 | 0 | 3.842395 | 0.718 | 0.002 | 0 | Mast cell |
| MARCO.1 | 0 | 3.814181 | 0.97 | 0.101 | 0 | Monocyte |
| C1QB.2 | 0 | 3.402157 | 0.995 | 0.176 | 0 | Monocyte |
| LYZ.2 | 0 | 3.315383 | 0.976 | 0.145 | 0 | Monocyte |
| S100A8.1 | 0 | 3.30724 | 0.879 | 0.098 | 0 | Monocyte |
| FCGR3A.1 | 0 | 3.269318 | 0.953 | 0.079 | 0 | Monocyte |
| IGLC3.1 | 0 | 8.555285 | 0.452 | 0.033 | 0 | Plasma cell |
| IGHG1 | 0 | 7.661731 | 0.67 | 0.032 | 0 | Plasma cell |
| IGLC1 | 0 | 7.619147 | 0.724 | 0.035 | 0 | Plasma cell |
| IGHG3 | 0 | 7.500602 | 0.571 | 0.041 | 0 | Plasma cell |
| JCHAIN | 0 | 6.01116 | 0.651 | 0.007 | 0 | Plasma cell |
| ACTA2.1 | 0 | 4.825018 | 0.865 | 0.135 | 0 | Smooth muscle cell |
| TAGLN.2 | 0 | 4.616783 | 0.956 | 0.379 | 0 | Smooth muscle cell |
| TPM2.2 | 0 | 4.381251 | 0.895 | 0.235 | 0 | Smooth muscle cell |
| MYH11 | 0 | 4.128628 | 0.728 | 0.042 | 0 | Smooth muscle cell |
| MYL9.2 | 0 | 3.654514 | 0.917 | 0.434 | 0 | Smooth muscle cell |
| CCL5 | 0 | 4.273499 | 0.658 | 0.017 | 0 | T cell |
| NKG7 | 0 | 4.03383 | 0.505 | 0.01 | 0 | T cell |
| CXCR4 | 0 | 3.650631 | 0.701 | 0.08 | 0 | T cell |
| IL32 | 0 | 3.563648 | 0.715 | 0.091 | 0 | T cell |
| CCL4.1 | 0 | 3.436351 | 0.508 | 0.163 | 0 | T cell |

**Table S5.  Marker genes identified by COSG for macrophage subclusters.**

|  | Macro_C0 | Macro_C1 | Macro_C2 | Macro_C3 | Macro_C4 | Macro_C5 | Macro_C6 | Macro_C7 |
| --- | --- | --- | --- | --- | --- | --- | --- | --- |
| 1 | F13A1 | MT-ND5 | FCER1A | OLR1 | FBLN1 | ITGBL1 | S100A12 | SPP1 |
| 2 | SLC40A1 | MT-ND4L | IL1R2 | LUCAT1 | FBLN2 | ITGB8 | PLAC8 | PLA2G7 |
| 3 | LYVE1 | MT-CYB | CD1C | TIMD4 | NOVA1 | TMEM196 | APOBEC3A | LPL |
| 4 | EMB | MT-ND1 | CLEC10A | TNF | MFAP5 | CLIC5 | LILRA5 | TM4SF19 |
| 5 | MAN1A1 | MT-CO2 | CD1E | IL1A | FBN1 | SOX5 | SERPINA1 | MATK |
| 6 | SELENOP | MT-CO3 | GPAT3 | IL6R-AS1 | ABCA8 | ZNF385B | FCN1 | CHIT1 |
| 7 | MRC1 | MT-ATP6 | HLA-DQB1 | CYB5D1 | CCN5 | CRTAC1 | SERPINB2 | SPOCD1 |
| 8 | EGFL7 | MT-ND3 | HLA-DQA1 | ITLN1 | PRELP | GABRA4 | RIPOR2 | MMP9 |
| 9 | GPR34 | MT-ND2 | LGALS2 | MIR155HG | CFH | CFI | FPR2 | MMP7 |
| 10 | MS4A4A | MT-CO1 | FLT3 | ABL2 | SFRP1 | STEAP4 | ADGRE1 | ASPHD1 |
| 11 | LGMN | MT-ND4 | PKIB | CCL3L1 | COL6A3 | TPD52L1 | TESC | MREG |
| 12 | CREG1 | MALAT1 | PLD4 | ACOT7 | AEBP1 | THBS4 | ITGAL | GPC4 |
| 13 | GYPC | MT-ND6 | JAML | LINC00346 | ITM2A | NTN4 | MCEMP1 | LINC02244 |
| 14 | CD209 | NKTR | ADAM28 | CCL4L2 | C3 | SEMA3E | CDA | FABP4 |
| 15 | FOLR2 | AC020916.1 | HLA-DPB1 | NCF1 | ANGPTL5 | GPR1 | ICAM3 | ITGA3 |
| 16 | RGL1 | XIST | CKLF | CCL20 | NEGR1 | ECRG4 | SELL | CYP27A1 |
| 17 | STAB1 | HNRNPH1 | HLA-DQA2 | THBD | PTGFR | HAS1 | RFLNB | MYOZ1 |
| 18 | WWP1 | IGKC | AREG | IL1B | DCLK1 | C10orf105 | SIGLEC10 | FCMR |
| 19 | MAF | NEAT1 | HLA-DPA1 | AZIN1-AS1 | PDGFRL | DLX3 | PROK2 | CHI3L1 |
| 20 | PMP22 | TTN | HCST | PDGFB | SPOCK1 | SORBS2 | ADGRE2 | LINC01010 |
| 21 | P2RY14 | N4BP2L2 | IL18R1 | PKP1 | PDGFRB | ENPP1 | RETN | SEZ6L2 |
| 22 | BLVRB | IGLC2 | CCR2 | EIF4E | GAS1 | SEMA5A | WARS | ACE |
| 23 | RNASE1 | PNISR | GAPT | FCGR3A | SLPI | GFPT2 | CD48 | AC078850.1 |
| 24 | COLEC12 | FTX | HLA-DRB1 | RBP4 | COL14A1 | MTUS2 | C19orf38 | DNASE2B |
| 25 | CTSC | STAB1 | TRAF1 | CXADR | LTBP1 | DIRC3 | SPN | FABP5 |
| 26 | MPEG1 | HNRNPA2B1 | ADAM8 | ALDH2 | COL6A1 | PCSK6 | P2RX1 | GOLGA7B |
| 27 | NRP1 | INTS6 | CLEC5A | CCL3 | PODN | DNASE1L3 | LILRA1 | ATP6V0D2 |
| 28 | C1QC | RNF213 | HLA-DRA | ACOD1 | GALNT15 | SGCA | SPATA13 | CAMK2B |
| 29 | FXYD6 | FUS | CIITA | CXCL3 | COL1A1 | DLX4 | HES4 | SLAMF9 |
| 30 | PLTP | TNFAIP2 | PHACTR1 | TNFAIP3 | MGST1 | UAP1 | FPR1 | RNF128 |
| 31 | PEPD | PRG4 | CTSH | CXCL2 | LAMA2 | ERRFI1 | CDC42EP2 | AF127936.2 |
| 32 | PDGFC | LUC7L3 | RTN1 | PLAUR | NTRK2 | TMEM100 | CD52 | RAB7B |
| 33 | SESN1 | JUN | HLA-DMA | NLRP3 | MXRA8 | SEMA3C | AQP9 | CADM1 |
| 34 | IQGAP2 | ARGLU1 | RGS2 | MIR222HG | CD248 | EML1 | ITGA4 | ALDH1A2 |
| 35 | WLS | SIGLEC1 | SAP30 | CCRL2 | SOD3 | SLC2A12 | SIRPB1 | TGM2 |
| 36 | DAB2 | CCNL2 | PID1 | IFI27 | SRPX | F5 | STXBP2 | PTCRA |
| 37 | SCN9A | TRA2B | LIMD2 | GAL3ST4 | MMP2 | CAV1 | FOLR3 | SYNGR1 |
| 38 | SLCO2B1 | TAGLN | CLEC4E | BCL2A1 | ECM2 | ADGRG2 | SYTL1 | RBP1 |
| 39 | TCN2 | DNM1 | RILPL2 | KDM6B | EBF1 | CRLF1 | EREG | RAMP1 |
| 40 | ME1 | MDM4 | HLA-DRB5 | ABCC3 | PRRX1 | IGFBP5 | CD300LF | CA2 |
| 41 | AKR1B1 | SRSF7 | GPR183 | SORL1 | ASPN | BTC | MXD1 | LINC01857 |
| 42 | HRH1 | RSRP1 | ATP2B1-AS1 | AL133330.1 | DPT | VCAM1 | AC018755.4 | TREM2 |
| 43 | SLC18B1 | HNRNPU | TSPAN33 | FERMT2 | IGFBP6 | SEMA3A | NRG1 | AC073611.1 |
| 44 | GNG2 | ADAP2 | IGFLR1 | AL031590.1 | SGCE | CDON | SLC2A6 | PRKAR1B |
| 45 | MCOLN1 | NXF1 | GRASP | FBP1 | CP | APOB | SCIMP | USP2 |
| 46 | C1QB | GARS-DT | CXorf21 | RALGDS | OLFML3 | SEMA6D | FCAR | TREML1 |
| 47 | GAS6 | LENG8 | AC023157.3 | GPR132 | C1S | AK1 | CD79B | P2RY11 |
| 48 | FUCA1 | ARHGEF1 | MNDA | CCL4 | VIT | RERG | G0S2 | DCSTAMP |
| 49 | C1QA | GOLGB1 | LAT2 | BHLHE40 | COL6A2 | SLC39A14 | PRAM1 | HAMP |
| 50 | IGF1 | HSP90AA1 | RPS29 | SLC16A10 | NID1 | NDUFA4L2 | SIGLEC14 | FAM20C |

**Table S6.  10 hub genes of all modules identified by hdWGCNA.**

| gene_name | module | kME | gene_name | module | kME |
| --- | --- | --- | --- | --- | --- |
| MYL6 | Mac-M1 | 0.572539 | DAB2 | Mac-M7 | 0.6591135 |
| GAPDH | Mac-M1 | 0.563678 | LILRB5 | Mac-M7 | 0.5186168 |
| TSPO | Mac-M1 | 0.553675 | A2M | Mac-M7 | 0.474211 |
| CLIC1 | Mac-M1 | 0.551026 | SIGLEC1 | Mac-M7 | 0.4710306 |
| PFN1 | Mac-M1 | 0.54877 | SCN9A | Mac-M7 | 0.447639 |
| CFL1 | Mac-M1 | 0.540829 | EMP1 | Mac-M7 | 0.4437115 |
| PSMA7 | Mac-M1 | 0.515741 | MAMDC2 | Mac-M7 | 0.4404713 |
| CHCHD2 | Mac-M1 | 0.503863 | GAS6 | Mac-M7 | 0.4402399 |
| ENO1 | Mac-M1 | 0.500603 | PEPD | Mac-M7 | 0.4370847 |
| ARPC1B | Mac-M1 | 0.486876 | TGFBI | Mac-M7 | 0.4370165 |
| C1R | Mac-M2 | 0.765788 | RNASE1 | Mac-M8 | 0.7388954 |
| C1S | Mac-M2 | 0.749471 | SELENOP | Mac-M8 | 0.7186064 |
| SCARA5 | Mac-M2 | 0.728014 | C1QA | Mac-M8 | 0.6869628 |
| COL6A2 | Mac-M2 | 0.723001 | C1QB | Mac-M8 | 0.6868359 |
| TNXB | Mac-M2 | 0.699842 | NPC2 | Mac-M8 | 0.6752605 |
| DCN | Mac-M2 | 0.691968 | FOLR2 | Mac-M8 | 0.6709127 |
| PLAC9 | Mac-M2 | 0.689668 | MS4A4A | Mac-M8 | 0.6660694 |
| MGP | Mac-M2 | 0.685322 | C1QC | Mac-M8 | 0.6623343 |
| TIMP3 | Mac-M2 | 0.674487 | FTL | Mac-M8 | 0.6570427 |
| LTBP4 | Mac-M2 | 0.671076 | PMP22 | Mac-M8 | 0.6561323 |
| MT-ATP6 | Mac-M3 | 0.929464 | FCN1 | Mac-M9 | 0.617629 |
| MT-ND3 | Mac-M3 | 0.917196 | CD48 | Mac-M9 | 0.6051508 |
| MT-CO1 | Mac-M3 | 0.908396 | SERPINA1 | Mac-M9 | 0.5694798 |
| MT-ND4 | Mac-M3 | 0.904909 | AREG | Mac-M9 | 0.5550057 |
| MT-CO3 | Mac-M3 | 0.899969 | LSP1 | Mac-M9 | 0.5310523 |
| MT-CO2 | Mac-M3 | 0.897003 | CD52 | Mac-M9 | 0.530745 |
| MT-CYB | Mac-M3 | 0.896551 | IL1R2 | Mac-M9 | 0.517152 |
| MT-ND1 | Mac-M3 | 0.863274 | CORO1A | Mac-M9 | 0.4747795 |
| MT-ND2 | Mac-M3 | 0.809431 | RILPL2 | Mac-M9 | 0.4726765 |
| MT-ND5 | Mac-M3 | 0.721941 | LIMD2 | Mac-M9 | 0.4644505 |
| PTPRC | Mac-M4 | 0.407557 | FOSB | Mac-M10 | 0.6766425 |
| ITGA4 | Mac-M4 | 0.407534 | EGR1 | Mac-M10 | 0.6750541 |
| TRAF3IP3 | Mac-M4 | 0.327235 | JUN | Mac-M10 | 0.6412508 |
| PTPRE | Mac-M4 | 0.311365 | IER2 | Mac-M10 | 0.6272242 |
| AKNA | Mac-M4 | 0.298183 | HSP90AA1 | Mac-M10 | 0.6121016 |
| CD69 | Mac-M4 | 0.289582 | DUSP1 | Mac-M10 | 0.609634 |
| RUNX3 | Mac-M4 | 0.281986 | DNAJB1 | Mac-M10 | 0.592717 |
| TUBA4A | Mac-M4 | 0.278177 | AC020916.1 | Mac-M10 | 0.5530083 |
| AC058791.1 | Mac-M4 | 0.264417 | DNAJA1 | Mac-M10 | 0.5331868 |
| SPATA13 | Mac-M4 | 0.256574 | JUND | Mac-M10 | 0.509024 |
| RPS12 | Mac-M5 | 0.761327 | LYZ | Mac-M11 | 0.529675 |
| RPLP1 | Mac-M5 | 0.759974 | TIMP1 | Mac-M11 | 0.4828077 |
| RPS14 | Mac-M5 | 0.754482 | LDHA | Mac-M11 | 0.4640293 |
| RPS3A | Mac-M5 | 0.753393 | GNAI2 | Mac-M11 | 0.457727 |
| RPL26 | Mac-M5 | 0.746497 | RPL36AL | Mac-M11 | 0.4422409 |
| RPL10 | Mac-M5 | 0.743742 | HLA-DRB5 | Mac-M11 | 0.3915877 |
| RPS15A | Mac-M5 | 0.74107 | MTDH | Mac-M11 | 0.3786047 |
| RPL21 | Mac-M5 | 0.740743 | HLA-DMA | Mac-M11 | 0.3772757 |
| RPS29 | Mac-M5 | 0.737084 | HLA-A | Mac-M11 | 0.3750662 |
| RPS23 | Mac-M5 | 0.736845 | RHOC | Mac-M11 | 0.3705376 |
| IL1B | Mac-M6 | 0.739088 |  |  |  |
| PLAUR | Mac-M6 | 0.709929 |  |  |  |
| NFKBIA | Mac-M6 | 0.706967 |  |  |  |
| IER3 | Mac-M6 | 0.694145 |  |  |  |
| CXCL2 | Mac-M6 | 0.68786 |  |  |  |
| OLR1 | Mac-M6 | 0.672559 |  |  |  |
| BCL2A1 | Mac-M6 | 0.662282 |  |  |  |
| TNFAIP3 | Mac-M6 | 0.662175 |  |  |  |
| SOD2 | Mac-M6 | 0.65209 |  |  |  |
| CXCL3 | Mac-M6 | 0.650721 |  |  |  |

**Table S7. Informations of OA patients and control subjects in our study**

| No. | Age | Gender | Joint | OA | Grade |
| --- | --- | --- | --- | --- | --- |
| 1 | 41 | M | Knee | No | - |
| 2 | 45 | F | Knee | No | - |
| 3 | 52 | M | Knee | No | - |
| 4 | 56 | F | Knee | No | - |
| 5 | 60 | F | Knee | No | - |
| 6 | 60 | M | Knee | No | - |
| 7  8  9  10  11  12 | 61  63  66  67  68  71 | M  M  F  M  F  F | Knee  Knee  Knee  Knee  Knee  Knee | No  No  No  No  No  No | -  -  -  -  -  - |
| 13  14  15  16  17  18  19 | 57  65  65  67  68  72  58 | F  M  F  F  M  M  M | Knee  Knee  Knee  Knee  Knee  Knee  Knee | Yes  Yes  Yes  Yes  Yes  Yes  Yes | 1-2  1-2  1-2  1-2  1-2  1-2  3-4 |
| 20 | 61 | M | Knee | Yes | 3-4 |
| 21 | 68 | F | Knee | Yes | 3-4 |
| 22 | 72 | F | Knee | Yes | 3-4 |
| 23 | 78 | M | Knee | Yes | 3-4 |
| 24 | 80 | M | Knee | Yes | 3-4 |

**Table S8. primer sequence**

| Gene | Sequence (5’ —— 3’) | |
| --- | --- | --- |
|  | Forward | Reverse |
| Il6 | TACATCAGCACCTCACAAGC | AGAAACAGTCCAGCCCATACT |
| Gapdh | GCTACCAAACTGGATATAATCAGG | GGACTCTGGCTTTGTCTTTCTT |
| Cxcl10 | GACACTGAGCAAGAGAGGCCCTA | TGGGATGGAAATTGTGAGGGA |
| Tnf | TCTCCATCACTCCCCTTTAC | TTCGGCAGTTACTTTTGTCTC |

**Table S9.  Top 50 proteins identified by immunoprecipitation-mass spectrometry (IP-MS).**

| Accession | Protein | PSMs | Classification | Decision | Functional Relevance to Oxidative Stress |
| --- | --- | --- | --- | --- | --- |
| U5LP42 | Anti-H5N1 hemagglutinin monoclonal antibody H5M9 heavy chain | 201 | Antibody contaminant | Exclude | No |
| A0A125T908 | Light chain kappa | 56 | Antibody contaminant | Exclude | No |
| P56480 | ATP synthase subunit beta, mitochondrial \| | 44 | Metabolic enzyme | **Retain** | No |
| G0YP42 | Anti-human Langerin 2G3 lambda chain | 102 | Antibody contaminant | Exclude | No |
| A0A2S0DFH4 | Anti-HIV fusion peptide monoclonal antibody light chain | 20 | Antibody contaminant | Exclude | No |
| A0A2S0DFI8 | Anti-HIV fusion peptide monoclonal antibody light chain | 18 | Antibody contaminant | Exclude | No |
| Q3TSB7 | Actin, cytoplasmic 2 | 36 | Abundant structural protein | Exclude | No |
| Q546G4 | Albumin | 37 | Abundant secreted protein | **Retain** | No |
| Q3TG92 | Actin alpha cardiac | 33 | Abundant structural protein | Exclude | No |
| Q3TIZ0 | Tubulin alpha-1C chain | 27 | Abundant structural protein | Exclude | No |
| P68369 | Tubulin alpha-1A chain | 26 | Abundant structural protein | Exclude | No |
| A8DUK4 | Beta-globin | 26 | Abundant intracellular protein | **Retain** | No |
| Q5FW97 | phosphopyruvate hydratase | 14 | Metabolic enzyme | **Retain** | No |
| Q64475 | Histone H2B type 1-B | 33 | Abundant nuclear protein | **Retain** | No |
| A8DUP7 | Beta-globin | 24 | Abundant intracellular protein | **Retain** | No |
| P99024 | Tubulin beta-5 chain | 17 | Abundant structural protein | Exclude | No |
| P10126 | Elongation factor 1-alpha 1 | 26 | Translation-related protein | **Retain** | No |
| P01797 | Ig heavy chain V-III region U61 | 20 | Antibody contaminant | Exclude | No |
| B2RY26 | Myosin-7 | 14 | Abundant structural protein | Exclude | No |
| A0A2R8VHP3 | Sodium/potassium-transporting ATPase subunit alpha | 40 | Abundant membrane protein | **Retain** | No |
| Q03265 | ATP synthase subunit alpha, mitochondrial | 22 | Metabolic enzyme | **Retain** | No |
| Q8BFU2 | Histone H2A type 3 | 35 | Abundant nuclear protein | **Retain** | No |
| P68372 | Tubulin beta-4B chain | 16 | Abundant structural protein | Exclude | No |
| Q8CD23 | Nucleolin | 14 | Abundant nuclear protein | **Retain** | No |
| Q4L0E7 | Type II cytokeratin Kb39 | 30 | Abundant structural protein | Exclude | No |
| Q9D8N0 | Elongation factor 1-gamma | 15 | Translation-related protein | **Retain** | No |
| A0A0G2JGX4 | Sodium/potassium-transporting ATPase subunit alpha | 10 | Abundant membrane protein | **Retain** | No |
| Q91VB8 | Alpha globin 1 | 18 | Abundant intracellular protein | **Retain** | No |
| E9Q557 | Desmoplakin | 16 | Abundant structural protein | Exclude | No |
| A0A075B5T3 | Immunoglobulin heavy variable 6-6 | 14 | Antibody contaminant | Exclude | No |
| Q9D6F9 | Tubulin beta-4A chain | 13 | Abundant structural protein | Exclude | No |
| P62806 | Histone H4 | 21 | Abundant nuclear protein | **Retain** | No |
| P53026 | Large ribosomal subunit protein uL1 | 20 | Abundant nuclear protein | **Retain** | No |
| **P08228** | **Superoxide dismutase [Cu-Zn] (SOD1)** | **14** | **Functional candidate protein** | **Retain (Target)** | **Yes** |
| Q3UV17 | Keratin, type II cytoskeletal 2 oral | 35 | Abundant structural protein | Exclude | No |
| A0A0A0MQF6 | Glyceraldehyde-3-phosphate dehydrogenase | 14 | Metabolic enzyme | **Retain** | No |
| Q3THW5 | Histone H2A.V | 26 | Abundant nuclear protein | **Retain** | No |
| Q543J5 | Antithrombin-III | 11 | Abundant secreted protein | **Retain** | No |
| E9Q5F6 | Polyubiquitin-C | 7 | Abundant protein | **Retain** | No |
| A0A1L1SQA8 | 40S ribosomal protein S25 | 13 | Abundant nuclear protein | **Retain** | No |
| Q8VDN2 | Sodium/potassium-transporting ATPase subunit alpha-1 | 6 | Abundant membrane protein | **Retain** | No |
| Q8C5B4 | Alpha-amylase | 11 | Abundant secreted protein | **Retain** | No |
| A2MXS5 | A6 anti-[4-hydroxy-3-nitrophenyl(Phenolate form)] acetyl mAb V-L region | 12 | Antibody contaminant | Exclude | No |
| Q54AH9 | Beta-2-globin | 12 | Abundant intracellular protein | **Retain** | No |
| Q80X85 | Small ribosomal subunit protein uS7m | 6 | Abundant nuclear protein | **Retain** | No |
| P63101 | 14-3-3 protein zeta/delta | 9 | Signalling protein | **Retain** | No |
| Q8C196 | Carbamoyl-phosphate synthase [ammonia], mitochondrial | 8 | Metabolic enzyme | **Retain** | No |
| Q3UDS0 | Heat shock cognate 71 kDa protein | 6 | Molecular chaperone | **Retain** | No |
| A0A0A6YWC8 | Vimentin | 12 | Abundant structural protein | Exclude | No |
| A2AKN8 | Major urinary protein 5 | 8 | Abundant secreted protein | **Retain** | No |
|  |  |  |  |  |  |

**Table S10.  Top 50 results of the virtual screening for potential compounds/ligands.**

| Ligand | Binding Affinity | Generic Name | Ligand | Binding Affinity | Generic Name |
| --- | --- | --- | --- | --- | --- |
| DB00390 | -7.5 | Digoxin | DB11273 | -6.9 | Dihydroergocornine |
| DB01220 | -7.4 | Rifaximin | DB16703 | -6.9 | Belumosudil |
| DB09297 | -7.4 | Paritaprevir | DB00288 | -6.8 | Amcinonide |
| DB11581 | -7.4 | Venetoclax | DB00549 | -6.8 | Zafirlukast |
| DB11614 | -7.4 | Rupatadine | DB00681 | -6.8 | Amphotericin B |
| DB11431 | -7.3 | Moxidectin | DB00696 | -6.8 | Ergotamine |
| DB01078 | -7.2 | Deslanoside | DB04868 | -6.8 | Nilotinib |
| DB01201 | -7.2 | Rifapentine | DB09280 | -6.8 | Lumacaftor |
| DB09027 | -7.2 | Ledipasvir | DB11574 | -6.8 | Elbasvir |
| DB11575 | -7.2 | Grazoprevir | DB11630 | -6.8 | Temoporfin |
| DB11691 | -7.2 | Naldemedine | DB00309 | -6.7 | Vindesine |
| DB12513 | -7.2 | Omaveloxolone | DB00619 | -6.7 | Imatinib |
| DB13345 | -7.2 | Dihydroergocristine | DB00826 | -6.7 | Natamycin |
| DB16098 | -7.2 | Atogepant | DB06210 | -6.7 | Eltrombopag |
| DB00762 | -7.1 | Irinotecan | DB06212 | -6.7 | Tolvaptan |
| DB01396 | -7.1 | Digitoxin | DB08995 | -6.7 | Diosmin |
| DB11613 | -7.1 | Velpatasvir | DB12026 | -6.7 | Voxilaprevir |
| DB15133 | -7.1 | Tepotinib | DB12457 | -6.7 | Rimegepant |
| DB00646 | -7 | Nystatin | DB12674 | -6.7 | Lurbinectedin |
| DB00966 | -7 | Telmisartan | DB15568 | -6.7 | Adagrasib |
| DB06290 | -7 | Simeprevir | DB15822 | -6.7 | Pralsetinib |
| DB11986 | -7 | Entrectinib | DB00773 | -6.6 | Etoposide |
| DB15328 | -7 | Ubrogepant | DB00895 | -6.6 | Benzylpenicilloyl polylysine |
| DB00872 | -6.9 | Conivaptan | DB04703 | -6.6 | Hesperidin |
| DB01609 | -6.9 | Deferasirox | DB08901 | -6.6 | Ponatinib |
